# Investigations into within- and between-subject resting-state amplitude variations

**DOI:** 10.1016/j.neuroimage.2017.07.014

**Published:** 2017-10-01

**Authors:** Janine Bijsterbosch, Samuel Harrison, Eugene Duff, Fidel Alfaro-Almagro, Mark Woolrich, Stephen Smith

**Affiliations:** a Centre for Functional MRI of the Brain (FMRIB), Wellcome Centre for Integrative Neuroimaging, Nuffield Department of Clinical Neurosciences, University of Oxford, UK; b Centre for Human Brain Activity (OHBA), Wellcome Centre for Integrative Neuroimaging, Department of Psychiatry, University of Oxford, UK

**Keywords:** Amplitude, Functional connectivity, Variability, Resting state

## Abstract

The amplitudes of spontaneous fluctuations in brain activity may be a significant source of within-subject and between-subject variability, and this variability is likely to be carried through into functional connectivity (FC) estimates (whether directly or indirectly). Therefore, improving our understanding of amplitude fluctuations over the course of a resting state scan and variation in amplitude across individuals is of great relevance to the interpretation of FC findings. We investigate resting state amplitudes in two large-scale studies (HCP and UK Biobank), with the aim of determining between-subject and within-subject variability. Between-subject clustering distinguished between two groups of brain networks whose amplitude variation across subjects were highly correlated with each other, revealing a clear distinction between primary sensory and motor regions (‘primary sensory/motor cluster’) and cognitive networks. Within subjects, all networks in the primary sensory/motor cluster showed a consistent increase in amplitudes from the start to the end of the scan. In addition to the strong increases in primary sensory/motor amplitude, a large number of changes in FC were found when comparing the two scans acquired on the same day (HCP data). Additive signal change analysis confirmed that all of the observed FC changes could be fully explained by changes in amplitude. Between-subject correlations in UK Biobank data showed a negative correlation between primary sensory/motor amplitude and average sleep duration, suggesting a role of arousal. Our findings additionally reveal complex relationships between amplitude and head motion. These results suggest that network amplitude is a source of significant variability both across subjects, and within subjects on a within-session timescale. Future rfMRI studies may benefit from obtaining arousal-related (self report) measures, and may wish to consider the influence of amplitude changes on measures of (dynamic) functional connectivity.

## Highlights

• We investigate variability in resting state amplitude between- and within-subjects.
• Clustering results reveal a distinction between cognitive and primary sensory/motor networks.
• Amplitudes in primary sensory/motor networks increase within subject towards the end of a scan.
• These increases in amplitude drive changes in apparent functional connectivity.
• Findings reveal complex relationships between amplitude and head motion.

## 1 Introduction

The Human Connectome Project (HCP) is a unique neuroimaging research resource, consisting of an extensive set of high quality imaging data from a large number of healthy subjects (Van Essen et al., 2013). For the first time, we have access to four repeat resting-state fMRI (rfMRI) scans per subject (a total of 60 min), from a very large group of study participants, alongside extensive demographic and behavioural subject measures. The combined availability of multiple long scans per subject, and a high number of subjects, offers a valuable opportunity to investigate and differentiate between within-subject and between-subject variability. Gaining a better understanding of the types of variability that we observe in rfMRI data across subjects, and whether or not we see the same types of variability within subjects over time, is important in relation to the biomarker potential of rfMRI. If the aim is to develop rfMRI to the point where it can be used on a single case basis for diagnosis, prognosis or individualised treatment, it is essential to differentiate between artifactual variability, within-subject (state) variability and between-subject (trait) variability.

Several studies have been published that use the wealth of between-subject information available in the HCP data. These studies have, for example, identified brain correlates of a positive-negative behavioural mode of population variation (Smith et al., 2015), and have showed that connectivity profiles can be used to predict fluid intelligence (Finn et al., 2015). However, analysing and interpreting such between-subject correlations is challenging, partly because many of the demographic measures of interest (including IQ and BMI) are also correlated with motion (Siegel et al., 2016). A recent study has revealed that the within-subject patterns of associations between functional connectivity and motion are very similar to the between-subject patterns of associations between functional connectivity and motion (Siegel et al., 2016). This suggests that subject head motion forms an important potential confound for correlational studies.

In addition to these types of between-subject correlational research, several studies have also investigated within-subject changes in rfMRI. Data acquired from the same individual subject over approximately 18 months has shown that within-subject variability of functional connectivity over time is especially high in visual and sensorimotor cortices, whereas the same is not true for between-subject variability (Laumann et al., 2015, Poldrack et al., 2015). The same dataset was also used to identify two different functional connectivity patterns (meta-states) that occurred repeatedly over time and were associated with significant differences in self reported levels of attention and tiredness (Shine et al., 2016). These findings point to the presence of significant variability within subjects over time. This type of within-subject variability is currently poorly characterized and understood, and may add a further confound to both between-subject correlational studies and to dynamic functional connectivity studies, that is commonly overlooked.

In this work, we focus primarily on the amplitudes of resting state BOLD signal fluctuations (i.e., the standard deviation of time series), because the amplitudes provide a localised summary measure for each resting state network that is relatively easy to estimate and interpret, and also has a direct, albeit complex impact on correlations between different regions’ timeseries (i.e., apparent functional connectivity) (Cole et al., 2016). The primary index of amplitude used in this paper is a measure of the relative size of BOLD fluctuations. This timeseries amplitude measure is closely related to the (fractional) amplitude of low frequency fluctuation (ALFF), which is a measure of low frequency power rather than of time series variance (Kannurpatti and Biswal, 2008, Zang et al., 2007, Zou et al., 2008). Previous work has linked between-subject variability in regional (f)ALFF to inter-individual difference in various aspects of behaviour, such as working memory, executive control and response inhibition (Mennes et al., 2011, Xu et al., 2014, Zou et al., 2013). Here, we extend this work by estimating associations between regional amplitude and a comprehensive set of measures including behaviour and lifestyle factors, subject head motion, and functional connectivity. We explicitly do not assume that the timeseries amplitude measure adopted in this work is driven exclusively by neuronal signal fluctuations (an assumption that is often made in the fALFF literature). In fact, we extensively test the influence of subject head motion on within and between subject variability in amplitude, as well as the indirect influence of head motion on functional connectivity estimates.

Changes in signal amplitude in either (or both) of two regions’ resting-state timeseries can result in changes in correlation (functional connectivity) between the two time series (Friston, 2011). For example, a change in correlation between two regions can be observed when a shared signal is added to both time series (leading to increased amplitude of both time series and increased correlation between them), or when an unshared signal is added to one of the time series (leading to increased amplitude in one of the time series and decreased correlation between the two time series). Therefore, many differences in functional connectivity that are observed between subject groups or within a subject across multiple scans may be explained by the existence of shared or unshared additive signals (Cole et al., 2016, Duff et al., 2017). Such additive signals can result from a variety of different sources, including: changes in neural processing, changes in non-neural noise sources, and changes in the local signal to noise ratio. For example, previous work has shown that differences in preprocessing strategies can significantly alter functional connectivity estimates (Gavrilescu et al., 2008, Weissenbacher et al., 2009). Therefore, understanding the variability in the amplitude of resting state networks plays an important role in functional connectivity more generally.

The aim of this work was to characterise between-subject and within-subject variability in resting state network amplitudes. We hypothesised that some aspects of variability are common both across subjects and within subjects (i.e., variability caused by state differences), whereas other types of variability may only be present across subjects, and not within subjects (i.e., variability caused by trait differences). We show that differences in the subjects’ arousal state can drive amplitude variability both across subjects and within subjects, particularly in visual, somatosensory, and motor networks. Additionally, we reveal a complex relationship between network amplitudes, behaviour and subject head motion.

## 2 Material and methods

### 2.1 Data

This study primarily uses data from the Human Connectome Project S900 release of resting state fMRI data from 819 subjects (452 male, mean age 28.8 ± 3.7 years old) (Van Essen et al., 2013). Each subject underwent a total of 4 resting state scans of 15 min duration over 2 days. Multiband echo planar imaging was used with an acceleration factor of 8 to achieve whole brain imaging at 2 mm isotropic resolution with a TR of 0.73 s (Moeller et al., 2010, Ugurbil et al., 2013).

In addition to HCP data, data from UK Biobank was used in order to replicate findings, and to perform between-subject correlations between BOLD signal amplitude and between-subject measures relating to arousal. Resting state scans (one per subject) were acquired using similar parameters to HCP for a duration of 6.10 min (2.4 mm spatial resolution, TR = 0.735 s, multiband acceleration factor of 8) (Miller et al., 2016). Data from 5847 UK Biobank subjects were used (2774 male, mean age 62.3 ± 7.5 years old).

### 2.2 Data pre-processing

The HCP data were preprocessed following HCP minimal preprocessing pipelines, containing tools from FSL, Freesurfer and HCP workbench (Fischl et al., 1999, Glasser et al., 2013, Jenkinson et al., 2012, Marcus et al., 2013, Smith et al., 2013a). ICA was performed for each run independently, and FIX (FMRIB's ICA-based X-noiseifier) was used to identify and regress out spatially structured noise components (Griffanti et al., 2014, Salimi-Khorshidi et al., 2014). Following spatial and temporal preprocessing, the data were in a grayordinate coordinate system that combines surface-based cortical regions and volumetrically represented subcortical regions (Glasser et al., 2013).

Biobank data preprocessing included correction for motion and distortions, high pass filtering, and FIX cleaning (Miller et al., 2016). The biobank data were analysed in volumetric space, as cortical modelling has not yet been applied to this huge dataset.

### 2.3 Group ICA and dual regression

For both HCP and UK Biobank data, temporal concatenation group ICA was performed to extract maps for 25 group-level ICA networks (and separately for 200 group-level ICA components in HCP data). The primary analyses presented in this work are based on the 25-dimensional group ICA results, because this dimensionality is commonly adopted in the literature and the resulting network structure closely matches commonly studied resting state networks (and can be easily matched between HCP and UK Biobank data by qualitative inspection). Multiple regression of these group ICA maps onto the rfMRI data from each run was performed to obtain time series for each resting state network for each run (1200 timepoints per run, 4800 timepoints in total per subject for the HCP data; 490 timepoints per subject for the Biobank data). Note that the post-processed HCP900 Parcellation + Timeseries + Netmats (PTN) data are publicly available (https://db.humanconnectome.org).

### 2.4 Amplitude estimation

In order to estimate the amplitude for each run (or for sub-blocks of each run), the temporal standard deviation across the run was calculated separately for each network (using the timeseries obtained from a multiple regression of the group ICA maps on the rfMRI data from each subject). Therefore, the standard deviation calculated based on the extracted timeseries reflects the voxel-wise standard deviation in spatial regions that contribute strongly to the group ICA maps. No variance normalisation was applied before calculating the amplitudes, as we were interested in the signal amplitudes and the within- and between-subject variability in this.

### 2.5 Canonical Correlation Analysis

For the CCA, we adopted an identical approach as described previously (Smith et al., 2015). Briefly, behavioural data were normalised and demeaned and confound variables (listed below) were regressed out. Implicit imputation was used to account for missing data in the behavioural variables. Similarly, the same set of confound variables was removed from the [subjects x ICA-networks] matrix of amplitudes. To avoid overfitting in the CCA, PCA was used as a dimension reduction step to reduce the behavioural measures matrix to size subjects x 100 (that is, keeping the top 100 subject-weight eigenvectors to feed into the CCA).

Confound variables that were removed from both the behavioural and amplitude data before performing the CCA included: acquisition reconstruction software version, subject motion, height, weight, systolic blood pressure, diastolic blood pressure, blood hemoglobin A1C, cube-root of total brain volume (including ventricles), cube-root of intracranial volume, and the squares of these (except for the acquisition reconstruction software), leading to a total of 17 confound variables.

CCA was performed (using canoncorr in Matlab) following: Y∗A=U∼X∗B=V; where X is the set behavioural measures, Y is the network amplitudes, and A and B are optimised such that the correlation between U and V is maximal (Hotelling, 1936). The correlation between the resulting pairs of subject weight vectors (one pair of U and V per CCA mode) indicates how strongly the mode of population covariation is represented in both the behavioural measures and the amplitudes. Significance of this correlation was estimated using permutation testing (n = 100,000), where the permutations kept the family (twin) structure in the data intact (Winkler et al., 2015).

In order to relate the CCA mode of population covariation to amplitude and behaviour, we performed post-hoc correlations between U and the amplitude matrix, and between V and the behavioural measures matrix. The CCA loadings for amplitudes can be found in Fig. 2 (red and magenta lines), and loadings for the behavioural measures are presented in Supplementary Fig. S2. The first significant CCA mode of population covariation explained at most 16.8% of variance in the behavioural measures (correct responses on the variable short Penn line test of spatial orientation), and at most 9.3% of variance in the amplitudes (IC 2; DMN). The second significant CCA mode explained at most 9.8% of variance in the behavioural measures (frequency of consuming 5 + alcoholic beverages in the heaviest 12-month period), and at most 36.9% of variance in the amplitudes (IC 18; cerebellum).

## 3 Results

### 3.1 Between-subject clustering analyses of BOLD amplitude

In order to obtain a better understanding of the way in which amplitudes of different resting state networks covary across subjects, we performed a clustering analysis. A correlation matrix (ICA components * ICA components) was calculated from the amplitude matrix (subjects * ICA components, obtained from concatenated timeseries across four runs), and Ward's clustering (using FSLnets, https://fsl.fmrib.ox.ac.uk/fsl/fslwiki/FSLNets) was performed to identify which resting state networks' amplitudes covaried across subjects in a similar way (Smith et al., 2013b). Note that this amplitude correlation matrix is estimated across subjects, as opposed to a functional connectivity matrix, which is calculated within a subject over time.

**Fig. 1:**
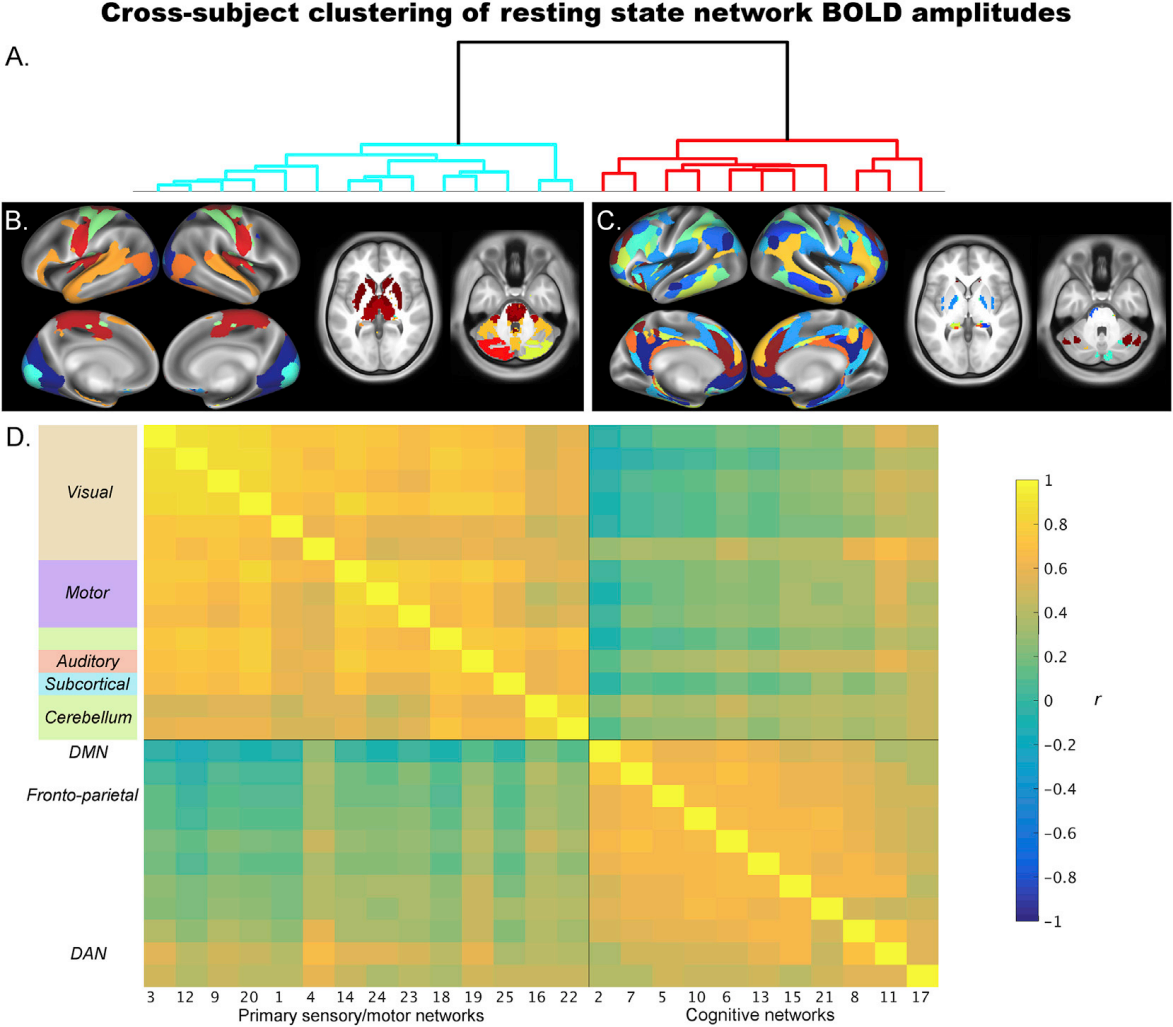
Between subject clustering of network amplitude correlation matrix. The cluster tree at the top (A) clearly separates the networks into two distinct clusters. For display here in B and C, each grayordinate was assigned to one of the 25 networks determined by the maximum value for the grayordinate across the group ICA maps. These “find-the-biggest” maps were then separated into two maps based on the cluster tree, to show the 14 primary sensory/motor networks on the left and the 11 cognitive networks on the right. The network x network between subject correlation matrix (*r*) is shown at the bottom (D) and reveals positive correlations within each of the two cluster groups (i.e., between networks in the primary sensory/motor cluster and between networks in the cognitive cluster).

Fig. 1 shows two clearly distinct clusters of RSNs (ICA components; optimal k = 2 out of k = 1–10 as determined using the Calinski-Harabasz criterion). Strong positive correlation is observed within each cluster (average z-transformed correlation within primary sensory/motor cluster 1 *z* = 0.84 ± 0.21; average z-transformed correlation within cognitive cluster 2 *z* = 0.67 ± 0.13), and lower correlation between the clusters (off-diagonal average z-transformed correlation between the clusters *z* = 0.28 ± 0.18). The first cluster contained 14 of the 25 networks, including visual networks (IC 3, 12, 9, 20, 1, 4), motor networks (IC 14, 24, 23), cerebellar and subcortical networks (IC 28, 25, 16, 22), and the language network (IC 19). The second cluster contained 11 of the 25 networks, including the DMN (IC 2), the dorsal attention network (IC 11), fronto-parietal network (IC 10), and salience network (IC 15). Given this general organisation, we will refer to the RSNs in cluster one collectively as ‘primary sensory/motor networks’, and the RSNs in cluster two as ‘cognitive networks’ in the remainder of this work.

In the results described above and in Fig. 1, the language network was part of the group of “primary sensory/motor” networks. However, this finding may be related to the dimensionality of the ICA decomposition that groups together language and auditory regions into the same network. When looking at a higher dimensionality (ICA 200), auditory and language components were separated. It is noteworthy that the main split at the higher dimensionality occurs between cortical and subcortical components (Supplementary Fig. S1). Nevertheless, the clustering results among cognitive regions replicated the 25-dimensionality results described above, showing a clear separation into primary sensory and motor versus cognitive networks. At the higher dimensionality, the three auditory components were grouped with the primary sensory/motor cluster, while the language component was grouped with the cognitive cluster.

Note that the cross-subject correlation between timeseries amplitude and fALFF (calculated as the ratio of low frequency power 0.01–0.1 Hz to power across the full frequency range) was on average *z* = 0.96 (range *z* = 0.55–1.43), and the correlation between amplitude and ALFF (calculated as the power in the frequency range 0.01–0.1 Hz) was on average *z* = 2.50 (range *z* = 1.99–3.19). This shows that all amplitude measures represent highly comparable aspects of the BOLD data.

### 3.2 Behavioural correlates of BOLD amplitude

**Fig. 2:**
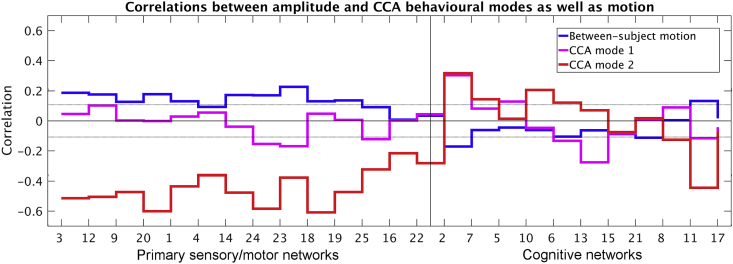
Correlations between resting state network amplitude, between subject motion and behavioural modes. For between subject amplitude-vs-motion correlations, root mean square relative (timepoint-to-timepoint) motion was averaged across each subject's 4 runs before calculating the between-subject correlation against network amplitude. For the between-subject correlations with the CCA modes of covariation, the subject-weight variables for the first two modes obtained from the CCA analysis were correlated against amplitudes. Dotted lines indicate the minimum correlation required to pass two-tailed significance testing (p < 0.05, Bonferroni corrected for multiple comparisons across 25 networks; |r| = 0.11).

To establish whether the temporal amplitudes of ICA-derived resting state networks contain behaviourally relevant between-subject information, we used Canonical Correlation Analysis (CCA) to reveal two significant modes of covariation (permuted P_corrected_<0.05). CCA is an approach that finds independent linear transformations for two sets of variables, such that the correlation between variables after transformation is maximised (for further details on the CCA please see the Material and Methods). In this instance, each significant mode of covariation represents a combination of behavioural measures that is maximally correlated with a combination of network amplitudes (Hotelling, 1936). Therefore, CCA results represent multivariate associations between lifestyle measures and amplitude measures. In essence, this analysis identifies a latent variable from a combination of amplitude measures that is most strongly associated with a latent behavioural variable (which represents a combination of different behaviour and lifestyle measures). The CCA weights (reported in Fig. 2 for amplitude measures and in Supplementary Fig. S2 for behavioural measures) represent how much each measured variable contributes to the latent variables identified by the CCA. CCA results can therefore be interpreted as significant correlations between brain and behaviour based on multivariate combinations of individual measurements. CCA has previously been used on HCP data to identify a positive-negative mode of population covariation (Smith et al., 2015).

Our CCA analysis identified two significant modes of covariation. The first was strongly negatively associated with items from the Achenbach Adult Self-Report Syndrome Scales (ASR), which assesses aspects of adaptive functioning (such as thought problems, withdrawal and aggression). This first CCA mode was positively linked to life satisfaction, conscientiousness and agreeableness (Fig. S2). The second mode of covariation was similar to the positive-negative mode previously reported from a smaller number (461) of HCP subjects (Smith et al., 2015) (correlation r = 0.56), and was positively linked to intelligence, and negative associated with smoking and drug use (Fig. S2).

Fig. 2 shows post-hoc correlations between the two significant modes of covariation and the original subject amplitudes across the ICA networks. This figure shows that the first mode of covariation was most strongly positively linked to DMN amplitude (IC network 2), while the second positive-negative mode was strongly negatively linked to primary sensory/motor amplitudes, and was also positively associated with DMN amplitude.

### 3.3 Within-subject amplitude changes across scans

**Fig. 3:**
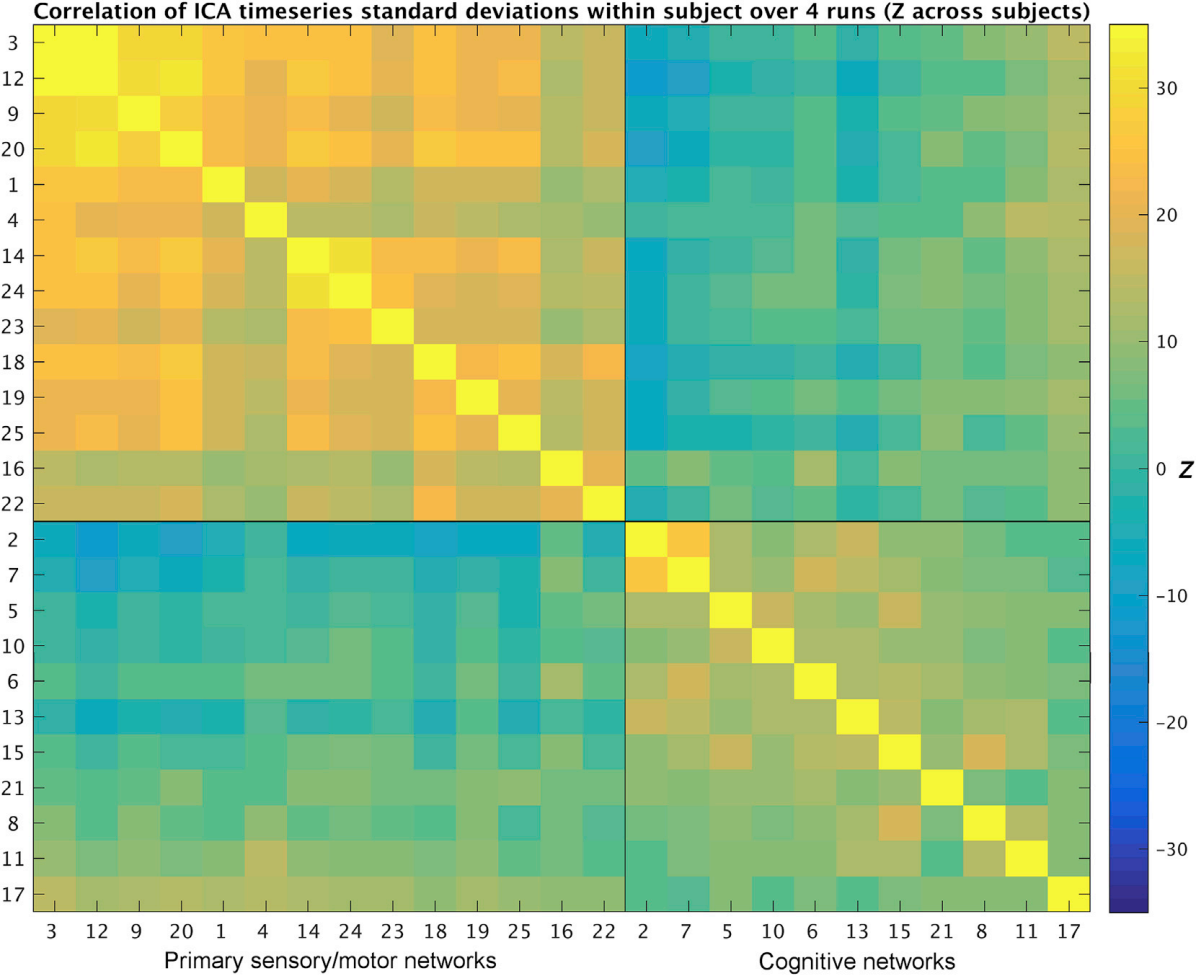
Within-subject replication of correlation matrix of network amplitude. One correlation matrix was estimated for each subject using the 4 different runs, this figure shows Z scores from a one group *t*-test performed across these subject correlation matrices. Similar results are obtained when splitting the subject data into 8 or 16 (sub-run) blocks rather than into the 4 original runs.

Next, we used the 4 runs that are available per subject in the HCP data in order to determine whether the same correlational pattern separating primary sensory/motor and cognitive networks can be observed within subjects. For each subject, ICA-derived resting state network amplitudes were estimated separately for each of the 4 resting state scans, resulting in a (networks x 4) matrix of amplitudes per subject. Correlating this with itself results in a (25*25 networks) correlation matrix estimated for each subject, that indicates which networks have a similar pattern of between-run amplitude variation to other networks. These subject correlation matrices were entered (element-wise) into a one-group *t*-test in order to combine correlation matrices across subjects. To directly compare the similarity between the between-subject and within-subject correlation matrices, networks were ordered according to the results of Ward's clustering analysis performed on the cross-subject correlation matrix shown in Fig. 1. Given that within-subject correlations were estimated separately within each subject, this analysis removes the mean amplitudes across subjects. Therefore, the results shows in Fig. 3 reveal state-dependent within-subject changes that occur over and above the between-subject findings reported in Fig. 1.

The results show that the pattern of correlations amongst network amplitudes that was found across subjects (Fig. 1), was largely replicated from this within-subjects amplitude correlations analysis (Fig. 3). However, the correlations between the amplitudes of cognitive networks within the bottom right block now appear weaker than the correlations between amplitudes of primary sensory/motor networks within the top left block. A paired across-subject *t*-test on the within-subject correlations averaged across primary sensory/motor networks vs. those averaged across cognitive networks (after Fisher's r-to-z transformation) confirmed that correlations between cognitive networks were significantly lower than correlations within primary sensory/motor networks t(819) = 19.9, p = 2*10^−72^. These findings suggest that state influences (which vary within subjects over time) influence correlations between amplitudes of primary sensory/motor networks. Note that state-dependent effects can lead to either increases or decreases in connectivity. Therefore, one possible explanation for the absence of state-dependent within-subject correlations between cognitive network amplitudes is that different cognitive mental states may engage different combinations of cognitive networks.

**Fig. 4:**
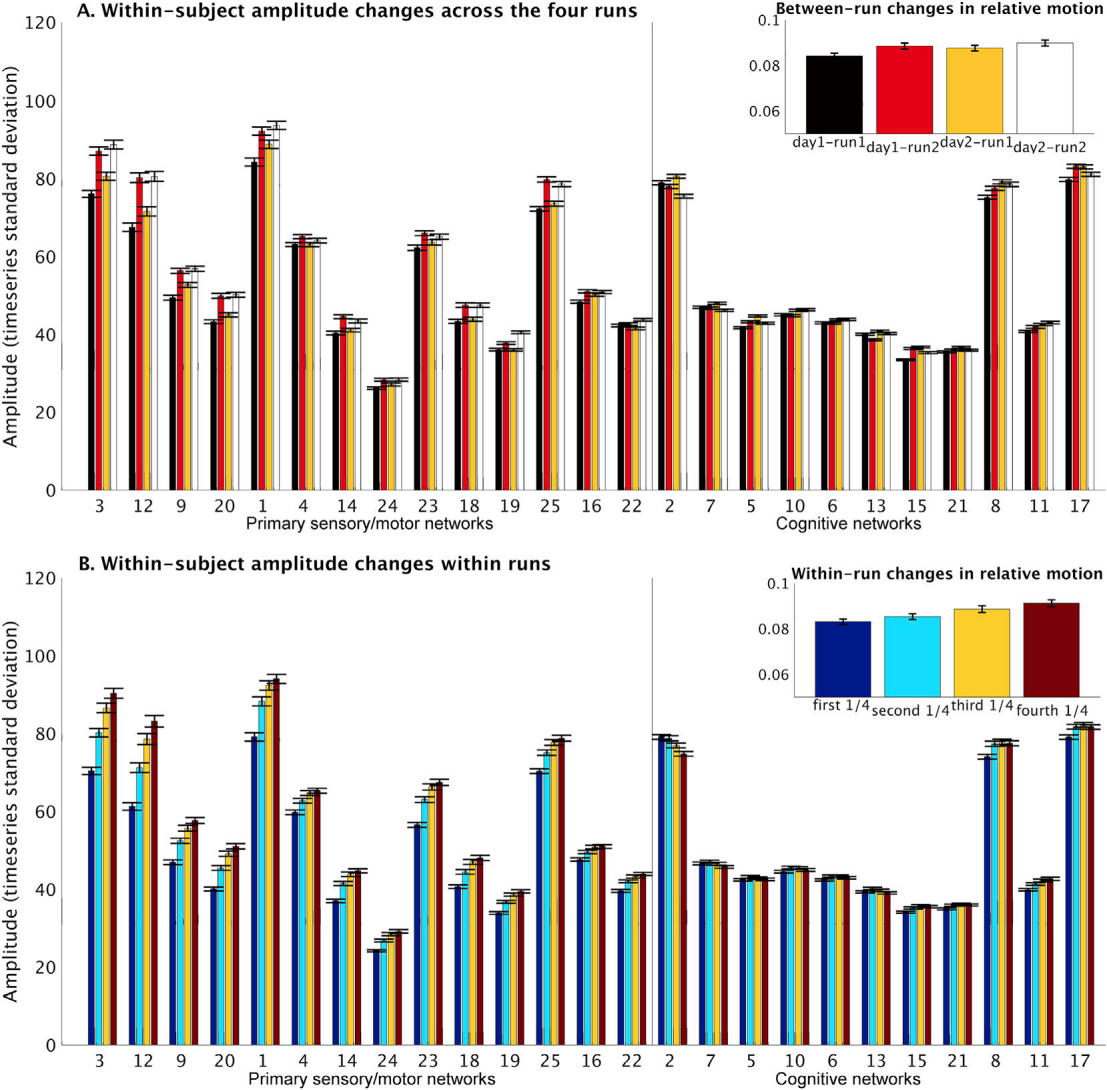
Within-subject variability in network amplitude. Figure A on the top shows amplitude changes across four runs, revealing that BOLD amplitudes in the primary sensory/motor networks were generally higher in the second scan compared with the first scan on both days. These finding cannot be explained by different phase encode directions for the runs (see supplementary results for more details). Figure B on the bottom shows amplitude changes within runs (across four 4-min blocks). The primary sensory/motor networks show a general increase in amplitude from the start of the run to the end, whereas a slight decrease in amplitude was observed in the DMN (IC 2; two-tailed t-tests showed that within-subject slopes were significant different from zero for each of the 4 runs: t_1_(818) = 10.0, p = 2.1*10^−22^; t_2_(818) = −7.1, p = 2.0*10^−12^; t_3_(818) = −8.8, p = 9.3*10^−18^; t_4_(818) = −3.6, p = 0.0003). The figure insets in the top right corner of A and B show changes in relative subject head motion between runs and within runs respectively. Error bars represent standard error of mean.

To look in more detail at the within-subject variability of network amplitudes, we considered amplitude difference across the four scans (obtained over two separate days), as well as gradual changes within each of the four 15-min scans. The results presented in Fig. 4A showed a consistent increase in the amplitude of all primary sensory/motor networks during the second scan of each day, compared with the first scan. Similarly, when looking at changes within each scan (over four 4-min blocks), a gradual increase in primary sensory/motor network amplitude can be observed over the course of the scans (Fig. 4B). Amplitude changes in cognitive networks were generally smaller and less consistent. Note that a similar pattern of within-run variability can be seen using fALFF as a measure of amplitude (Fig. S3).

Given that all primary sensory/motor networks showed strikingly similar patterns of amplitude change, we next performed statistical comparisons of these effects using a repeated measures ANOVA with within-subject factors for run (2 levels) and for day (2 levels). For the analysis of between-run changes, the inputs to the ANOVA were the amplitude calculated separately for each run, and averaged across the 14 networks in the primary sensory/motor cluster. As shown in Supplementary Fig. S4, the results reveal significant main effects of both run (*F*(1,818) = 271.7, *p* = 6.3*10^−53^) and day (*F*(1,818) = 6.4, *p* = 0.01), and a significant interaction effect between day and run (*F*(1,818) = 12.1, *p* = 0.001). The main effect of run represents a significantly higher amplitude in run 2 (mean 59.4) compared with run 1 (mean 54.8), and the main effect of day is driven by an overall increased amplitude on day 2 (mean 57.6) compared to day 1 (mean 56.6). Post-hoc paired comparisons showed that there was a significant difference between day 1 and 2 for run 1 (*t*(818) = -4.2, *p* = 3.0*10^−5^), but not for run 2 (*t*(818) = -0.5, *p* = 0.62). One possible interpretation of these findings is that subjects’ arousal levels may be higher at the start of day 1, compared with the start of day 2, due to the novelty of the scanner environment. For the analysis of within-run changes, we calculated the amplitude of each network for each 300 timepoints, and subsequently estimated the slope across the resulting 4 amplitude estimates per scan. These slopes were averaged across the 14 networks in the primary sensory/motor cluster and entered into an ANOVA with within-subject factors for run and day. For the analysis of within-run changes, we found significant main effects of both run (*F*(1,818) = 58.5, *p* = 5.6*10^−14^) and day (*F*(1,818) = 45.6, *p* = 2.8*10^−11^), but the interaction between day and run was not significant (*F*(1,818) = 0.08, *p* = 0.78). The main effect of run was driven by an increased slope in run 1 (mean 3.7) compared with run 2 (mean 2.8), and the main effect of day reflects a stronger slope on day 1 (mean 3.6) compared with day 2 (2.9), as can be seen in Supplementary Fig. S4.

To investigate the changes in BOLD amplitude from the start to the end of a scan at a whole-brain level, a temporally-resolved grayordinate-wise analysis was performed. At each grayordinate and every time point individually, a data vector was created by combining the normalised values from the BOLD timeseries from every subject and every run, resulting in a vector with 3280 datapoints (820 subjects*4 runs). The BOLD data amplitude was estimated by taking the standard deviation over this data vector (in effect, estimating the intensity standard deviation, pooling across subjects). By collapsing over subjects and runs in this manner, we can investigate fine-grained variance changes over the scan in a spatio-temporally resolved fashion. To create a summary measure of these variance changes, PCA was performed across the resulting [grayordinates x time points] BOLD data amplitude matrix. Prior to this, the first 12 timepoints were discarded because they were affected by magnetisation effects, and the overall amplitude timeseries for each grayordinate was normalised.

**Fig. 5:**
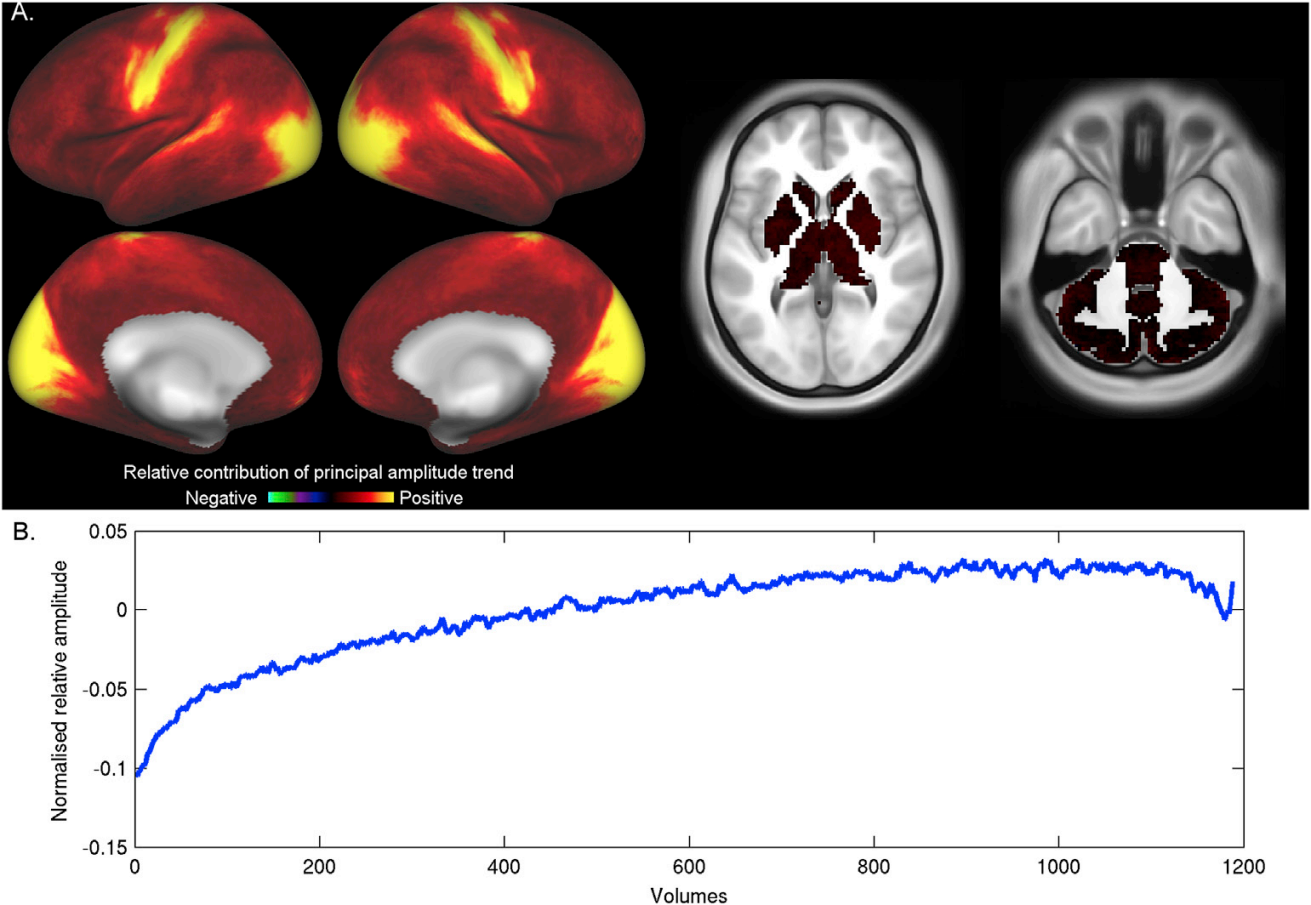
The first principal component of variance changes over time displays the characteristic increase in amplitude from the start to the end of the scan (temporal weights shown in panel B). The spatial weights for this principal component are shown in panel A, and demonstrate that this effect is mostly localised to the primary visual and primary sensorimotor regions.

The first component resulting from the PCA shows the pattern of increased variance across the duration of the scan (Fig. 5b, similar pattern to Fig. 4b). The resulting weight map (Fig. 5a) shows that the increases in BOLD amplitude are relatively unique to the primary sensory/motor cortices. Note that this map reveals very similar spatial structure to the regions that show increased amplitude during sleep relative to wakefulness (Fig. 3 in (Horovitz et al., 2008)). It is also noticeable that the weights in the postcentral gyrus (i.e., the primary somatosensory cortex), are higher than the weights in the precentral gyrus (primary motor cortex).

### 3.4 Role of subject motion

There is a correlation (across each of the 4 runs: r > 0.2, P_Bonferroni_<10^−8^) between subject head motion and amplitude, consistently seen across all primary sensory/motor networks (as shown in Fig. 2). This is confirmed by the significant change in subject head motion across the four resting state runs, and also across four 4-min blocks within a run (inserts at the top right side of Fig. 4). While the amount of variance in network amplitude explained by motion was relatively low (4%), these findings together suggest that changes in network amplitude might be linked to motion artifacts. In order to examine this possibility further, we performed three tests described below.

To determine whether the increase in amplitude was more likely to reflect BOLD signal of neuronal origin, or artifacts present in the measured dataset, we firstly looked at changes in the frequency spectra within each 15-min scan. Specifically, the area under the curve for the lowest (<0.08 Hz) and the highest (>0.1 Hz) frequencies were estimated based on the Fourier spectra of each 300 TR section of each scan. The results show an increase in low frequency power over the course of the scans for all of the primary sensory/motor networks, compared with smaller and more inconsistent changes in high frequency power (Fig. S5). While the low-frequency nature of amplitude changes may suggest a neuronal origin, artifacts such as gradual head motion, aliased physiological pulsation, and slow variations in respiration or heart rate are also known to influence the BOLD signal in the low frequency range. All data used in this work underwent extensive cleanup designed to minimize the potential influence of such sources of structured noise (including the removal of ICA components labelled as noise and of extended motion parameters). Nevertheless, to further exclude the possibility that changes in primary sensory/motor network amplitudes were purely driven by subject head motion, we perform two further tests described below.

**Fig. 6:**
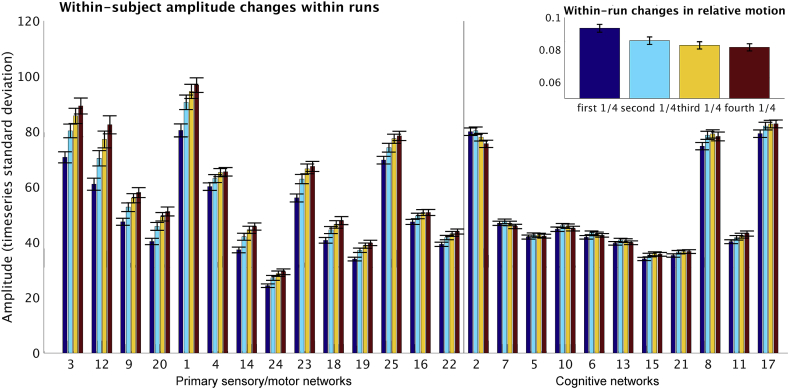
Increased primary sensory/motor amplitude is also observed in subjects that show decreases in head motion (as shown by figure insert at the top right).

To further determine whether the observed increase in primary sensory/motor network amplitude occurs as a result of increased motion, we selected a group of 164 subjects who most strongly displayed a pattern of decreased motion from the start of the run to the end of the run (i.e., the opposite pattern of the overall group average, which shows increased motion). In order to identify this subgroup of subjects, root mean square relative motion was calculated for each 4-min block, and the slope across 4 blocks was estimated separately for scan 1 and scan 2 obtained on day one. The slopes were averaged for each subject across the two scans, resulting in a single value that described the trend of within-run motion changes observed for each subject. The 164 subjects with the smallest slope were used for this analysis (mean slope −0.0039, range: −0.0133 to −0.0015). The results of the amplitude changes within run in these 164 subjects show that the same pattern of increased amplitude over the course of the scan was present in all primary sensory/motor regions in this group of subjects, despite a decrease in head motion over the course of the scans in this subject group (Fig. 6).

As a third and final test to determine the potential influence of motion on the amplitude findings presented here, we applied volume censoring to the data prior to calculating the amplitudes. Volume censoring was performed using a threshold of framewise displacement>0.2 and involved removing all of the supra-threshold volumes as well as one volume immediately preceding and two volumes following each supra-threshold motion spike (Power et al., 2013). Framewise displacement was estimated as the root mean square relative motion, and scans with fewer than 20% of volumes (<240) were excluded, leading to the removal of 50 scans. Volume censoring was performed separately for scans 1 and 2 on day one of the HCP data, and amplitudes were calculated separately. On average, 1082 vol were retained for each scan (range: 240–1200). Volume censoring has previously been shown to dramatically reduce motion-related effects observed in resting state fMRI data (Power et al., 2012a, Power et al., 2015).

The results show that after performing volume censoring to further remove effects of motion on the data, the increase in BOLD signal amplitudes in primary sensory/motor regions in the second scan compared to the first scan is still just as evident (Fig. S6). Given that the findings do not change as a result of volume censoring, and because amplitude estimates from censored data are potentially biased (i.e., estimates are less accurate if more volumes were removed during censoring), this step was not applied for the other results presented in this paper. This finding, taken together with the results presented above, strongly suggests that the observed increases in primary sensory/motor network amplitude do not occur as a direct artifactual result of increased head motion.

### 3.5 Differences between within-subject and between-subject variability

**Fig. 7:**
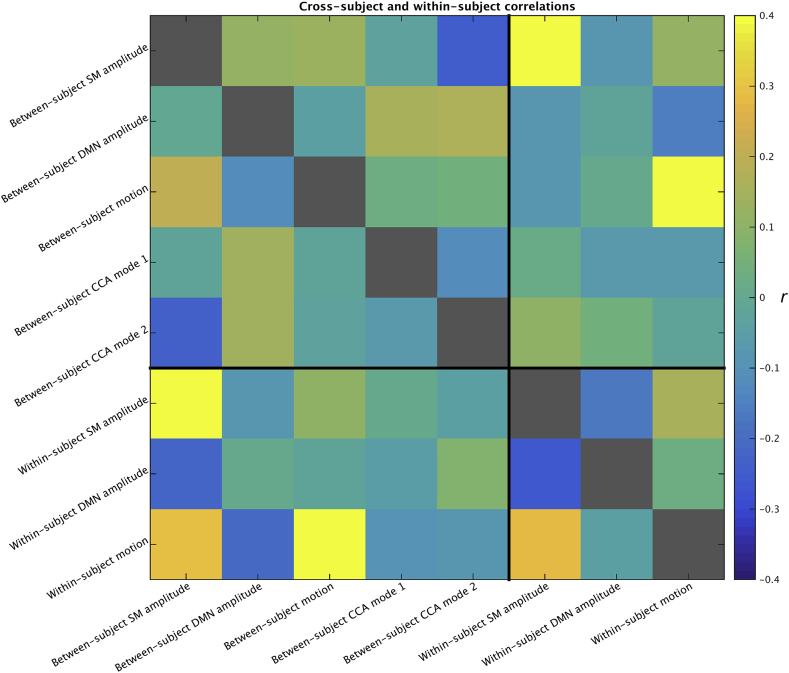
Associations between primary sensory/motor and default mode amplitude, motion and behavioural CCA modes of covariation. Within subject amplitudes and motion were estimated by calculating the slope with each run across 4 blocks and averaging the slopes across runs. Below the diagonal the full correlations between measures are shown, and above the diagonal the partial correlations. Six nuisance variables were regressed out of all elements first (height, weight, intracranial volume and the squares of these three).

In order to determine whether the within-subject associations between primary sensory/motor amplitude, DMN amplitude and motion mirrored the between-subject associations, we performed a direct comparison. Summary measures of amplitude and motion were extracted both within and between subjects as described below. Full and partial correlations of the between-subject and within-subject summary measures were performed (Fig. 7).

A summary measure of primary sensory/motor amplitude for each subject was calculated by averaging amplitudes across all 14 IC networks that clustered together (Fig. 1). Instead of averaging across cognitive networks, we used DMN amplitude (IC 2 only), because the correlations between cognitive networks is less pronounced within subjects (Fig. 3), and therefore averaging may not be as appropriate. Additionally, the DMN was the only network to show within-subject changes in amplitude, and is therefore of interest for this direct comparison between within and between subject associations. Averaged root mean square relative motion was used as a single value summary measure per subject. Two further between-subject summary measures were obtained from the behavioural measures based on the CCA results. Given that motion was regressed out of the input to the CCA (along with other nuisance variables), the behavioural modes of covariation obtained directly from the CCA are orthogonal to motion. To obtain summary behavioural variables that are not orthogonal to motion, we took an approach analogous to dual regression: the subject weights vector obtained from the CCA analysis for each of the two modes of covariation was regressed into the behavioural measures, and the resulting beta values were subsequently regressed into the (non-adjusted) behavioural measures. This approach provided two vectors for each of the significant behavioural modes of covariation.

For the within-subject comparison, the same measures described above for primary sensory/motor and DMN amplitude and for motion were calculated for four 4-min blocks of data within each scan. Subsequently, the slopes across the four blocks were calculated for each scan and for each measure (i.e., primary sensory/motor amplitude, DMN amplitude and RMS motion). These slopes were subsequently averaged across the two scans performed on day one. For the within-subject measures, these averaged slopes were correlated.

The results shown in Fig. 7 highlight a few key differences between within and between subject variability.

Firstly, a negative correlation was found between primary sensory/motor and DMN amplitude within subjects (r = −0.25, P_Bonferroni_ = 3.4*10^−13^), but not between subjects (r = −0.004, P_Bonferroni_>0.9). This is consistent with the result that between-subject primary sensory/motor amplitude is highly correlated with within-subject slope of primary sensory/motor amplitude (r = 0.55, P_Bonferroni_ = 1.7*10^−66^), but the same association was not found between the slope of within-subject DMN amplitude and between-subject DMN amplitude (r = 0.0021, P_Bonferroni_>0.9). This suggests that subjects who show the biggest increase in primary sensory/motor amplitude over the course of a scan, are also subjects with the highest overall primary sensory/motor amplitude, but no similar association holds for DMN amplitude.

Secondly, a negative correlation between between-subject DMN amplitude and between-subject motion was observed (r = −0.12, P_Bonferroni_ = 0.007), that was not present between within-subject slope of the DMN amplitude and within-subject slope of motion (r = −0.04, P_Bonferroni_>0.2). Primary sensory/motor amplitude was significantly correlated with motion both within subjects (r = 0.28, P_Bonferroni_ = 3.6*10^−14^), and between subjects (r = 0.21, P_Bonferroni_ = 2.4*10^−9^).

### 3.6 Role of arousal

Given the length of the resting state scans in the HCP (i.e., two scans of 15 min, back to back), it is highly likely that subjects are getting drowsy or falling asleep towards the end of the scans (despite instructions to stay awake and fixate). Previous research has shown that the probability of subjects still being awake drops to 0.5 after 10 min of scanning (Tagliazucchi and Laufs, 2014), and earlier findings have shown a relationship between light sleep and BOLD amplitude in the sensorimotor cortices (Horovitz et al., 2008, Laumann et al., 2015).

We used UK Biobank data to investigate if a relationship exists between sleep quality or quantity and primary sensory/motor network amplitude. The UK Biobank data contains many more subjects and a more comprehensive battery of self-report measures including a set of sleep-related items compared with the HCP data completed during the imaging visit (see Supplementary Methods for further information).

Firstly, the clustering analysis was repeated independently on the UK Biobank data (by performing Ward's clustering on the network * network correlation matrix that was calculated from the amplitude data for all subjects). The results replicated the separation of two clusters seen in the HCP data, where one of the clusters contains 11 primary sensory/motor components and the other contains 14 cognitive networks (Fig. S7). It is noticeable that the correlations between primary sensory/motor networks and cognitive networks (i.e., in the off-diagonal block on the bottom left) were somewhat higher in the UK Biobank data than in the HCP data. A potential reason for this may be the age of the participants (as UK Biobank subjects are drawn from an older population in comparison to the HCP subjects).

Next, we performed a between-subject correlation analysis between primary sensory/motor amplitude, DMN amplitude, subject head motion and self-report measures linked to sleep. As described above, amplitudes were averaged within the 11 primary sensory/motor networks based on the clustering results to calculate mean primary sensory/motor amplitude. Correlations were performed between averaged primary sensory/motor amplitude, DMN amplitude, subject head motion and a small set of 7 specific sleep-related self-report measures (snoring, daytime napping, daytime dozing, sleeplessness, sleep duration, sleep morning, and sleep chronotype).

There was a significant negative correlation between primary sensory/motor amplitude and sleep duration (r = −0.11, p = 8*10^−16^), but not between DMN amplitude and sleep duration (r = −0.03, p = 0.07). These findings are in line with the hypothesis that arousal may be linked to primary sensory/motor network amplitude, but not to DMN amplitude (Horovitz et al., 2008, Tagliazucchi et al., 2013, Tagliazucchi and Laufs, 2014). Although, note that these results are based on associations with self-report data regarding sleep patterns. It is possible that these self-report data may not be an accurate measure of sleep, or that other factors could drive both the amplitude and the self-report measures. All of the other correlations with sleep measures were below |r| = 0.1.

The between-subject correlation between primary sensory/motor network amplitude and motion was not replicated in the UK Biobank dataset (r = −0.02, p = 0.19). This may be a result of the shorter scan duration (15 min in HCP versus 6 min in Biobank), which may help subjects avoid falling asleep in the UK Biobank dataset compared with the HCP dataset. There was, however, a correlation between DMN amplitude and head motion (r = −0.13, p = 2*10^−22^).

### 3.7 Effect of amplitude changes on functional connectivity analyses

A question that is relevant to many functional connectivity studies is what effects the observed changes in BOLD signal amplitude have on connectivity analyses. In order to address this question, paired t-tests were performed to compare the network connectivity matrices between scan 1 and scan 2 of the HCP data acquired on the same day. Given that these scans are performed on the same subjects and in the same scanning session, we might expect that only minimal changes in functional connectivity would occur.

**Fig. 8:**
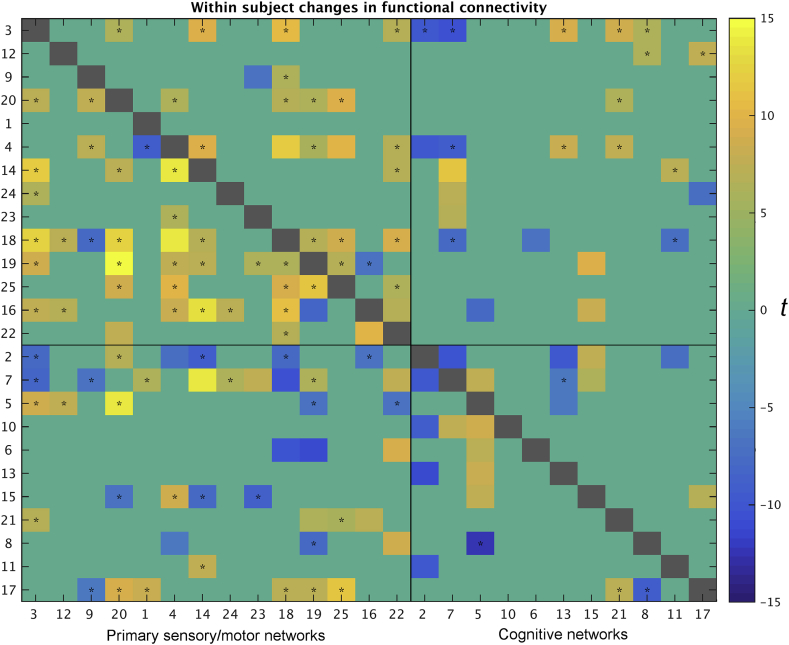
Changes in functional connectivity occurring as a result of amplitude changes. The colour scale reflects t statistics for the paired *t*-test between runs 1 and 2 on the same day (only edges that pass Bonferroni correction and have a |t|>6 are shown). Results for day 1 are shown below the diagonal, and results for day 2 above the diagonal. All of these changes in functional connectivity could be explained by changes in network amplitude. Cases where additive signal common to both networks explained the change in functional connectivity are marked *.

Using the dual regression time series from the 25 ICA networks, full connectivity matrices were estimated for each subject and for each run, and were z-transformed. Paired t-tests were performed separately for day 1 and day 2, and changes in functional connectivity between pairs of networks was determined significant if the paired *t*-test results passed Bonferroni correction (p < 1.6667*10^−4^), and the t-statistic was higher than 6. The results show many widespread and highly significant changes in edge strength within subjects between the first and the second scan of the day (Fig. 8).

The consistent increases in primary sensory/motor amplitude (Fig. 4) suggest that the changes in functional connectivity may be produced by simple changes in signal strength of certain regions. To assess this, we performed an Additive Signal Change analysis (ASC). This approach models the timeseries from each network as a linear combination of separate baseline and additive signals, and determines whether observed changes in functional connectivity between a pair of networks can be explained simply by the introduction of additional signal in one of two runs (Duff et al., 2017). Further details on the ASC analysis can be found in the Supplementary Methods. The approach is related to the conjunction method of Cole et al. (2016), but directly tests whether observed changes in amplitude are adequate to fully explain the changes in correlation. Briefly, an ASC analysis is performed by testing all possible signals that could be producing observed increased amplitude in one run. These signals are defined by their correlation with existing signal, with the constraint that these signals are not anti-correlated with this signal. Each such signal has a specific effect on correlation, such that it can be determined whether the observed signal fits into this range. These putative additive signals producing amplitude changes could reflect many types of changes, including changes in the amplitude of measurement noise, blood perfusion, or neural activity. In the present experiment, this analysis was used to determine whether observed changes in correlations might be wholly explained by differential changes in the amplitude of network signals, reflecting extensive changes in amplitude observed across the two scans reported above.

The results of the ASC analysis showed that all changes in functional connectivity could be explained by additive signals (i.e., changes in network amplitude). Additive signal common to both networks explained many of the changes in connectivity (as highlighted by * in Fig. 8), particularly between pairs of primary sensory/motor regions. The other significant changes in functional connectivity could all be explained by a mixture of common and unshared additive signals. Note that the changes in functional connectivity on day two are sparser than on day one. It is possible that the significant difference in amplitude between run 1 on day 1 and run 1 on day 2 (Supplementary Fig. S4) may be related to this. When comparing run 1 on days 1 and 2 directly in terms of functional connectivity, a small number of significant differences were identified, but only some of these differences could be explained by changes in amplitude (see Supplementary Fig. S8). These findings highlight the extent to which changes in amplitude can drive functional connectivity results.

## 4 Discussion

The amplitudes of spontaneous fluctuations in brain activity may be a significant source of variability which is commonly overlooked, despite its direct relevance to measures of functional connectivity. Here we investigated resting state amplitudes in two large-scale studies (HCP and UK Biobank), with the aim of determining between-subject and within-subject variability. Gaining a better understanding of the types of variability that exists across subjects and across runs within subjects is essential for successful normative and biomarker research in this age of larger sample sizes and population-based research.

Our findings showed a between-subject clustering that separated resting state networks into two clusters based on their amplitudes. The first cluster (of brain regions/networks whose amplitude variation across subjects were highly correlated with each other) grouped together visual, auditory, motor and somatosensory cortices, in contrast to a second cluster that contained cognitive networks such as the DMN, DAN, and frontoparietal network. These clustering results suggest that, for example, subjects with high BOLD amplitudes in the DMN would also show higher amplitudes in other cognitive networks. This separation of cognitive and primary sensory and motor regions based on BOLD amplitudes is consistent with previous work (Zhang et al., 2011). This between-subject covariation pattern was behaviourally relevant; the second mode of population covariation shown in Fig. 2 revealed that subjects with lower BOLD amplitudes across all of the primary sensory/motor networks (i.e., in the first cluster), scored more highly on positive behavioural measures such as intelligence. Hence, between-subject variability in resting state amplitude is of behavioural relevance, and distinguishes between cognitive and primary sensory/motor regions.

A similar separation of primary sensory/motor versus cognitive networks was found when correlations among amplitudes were calculated based on different runs within the same subject. Importantly, a within-subject change in amplitudes over time was observed reflecting an increase in amplitude from the start of the scan to the end of the scan (and also an increase in average amplitude during scan 2 compared with scan 1 acquired on the same day). This pattern was consistently seen across all primary sensory/motor networks, but was not present in any of the cognitive networks (Fig. 4). The observed increase in primary sensory/motor amplitudes towards the end of the scan was characterized by increased power in the low frequency range, suggesting that these findings cannot be explained by simple artifacts. Similar increases in low frequency power have been observed within subjects in a single scanning session previously (Duff et al., 2008). While there was a correlation between primary sensory/motor amplitude and subject head motion both within and between subjects, the increase in amplitude from the start to the end of the scan was still present after volume censoring the data, and was also observed in subjects who showed a decrease in motion from the start of the scan to the end of the scan. As such, the change in primary sensory/motor amplitudes over time was not simply driven by artifactual motion effects.

One possible reason for the increase in primary sensory/motor network amplitudes over the course of the scans could be a decrease in arousal as subjects become increasingly sleepy over the course of the scans. We tested the relationship between primary sensory/motor network amplitudes and arousal using between-subject sleep-related data available in the UK Biobank, and found a negative relationship between primary sensory/motor amplitude and sleep duration. This is consistent with previous studies that identified increases in rfMRI BOLD signal fluctuations during light sleep in visual and primary sensory/motor regions (Horovitz et al., 2008, Tagliazucchi et al., 2013, Tagliazucchi and Laufs, 2014), and with the finding that high within-subject variability in scans obtained across 18 months was particularly localised to visual and primary sensory and motor cortices (Laumann et al., 2015). Additionally, it has also been shown that differences in vigilance are reflected in the amplitude of the mean whole-brain signal (‘global signal’), which shows a spatial distribution that is very similar to Fig. 5 (Wong et al., 2012, Wong et al., 2016, Wong et al., 2013). Previous work has shown that high self-reported ‘sleepiness’, as measured directly after resting state fMRI scans, was significantly associated with increased functional connectivity in visual and sensorimotor networks (Stoffers et al., 2015), consistent with the increased connectivity between primary sensory/motor regions reported here (Fig. 8). These findings suggest that the subjects' arousal levels are a source of significant variability both across subjects and within subjects over time, and may be a confounding variable for group and session comparison studies. This role of arousal has not been much considered in rfMRI research, but future studies may wish to obtain a (self report) measure of arousal to be used as a covariate of no interest. For example, it might be worth asking subjects how many hours they slept the night before the scan, how tired they were feeling during the scan, and whether or not they got drowsy or fell asleep during the scan (Diaz et al., 2013).

It is possible that the changes in BOLD amplitude across subjects and within subjects over the course of a resting state scan may be partly linked to changes in physiological processes. Previous research has shown significant associations between cerebrovascular reactivity (i.e., the BOLD response to CO_2_ modulations) and both BOLD amplitude and ALLF (Golestani et al., 2016, Kannurpatti and Biswal, 2008). Light sleep is associated with marked changes in physiological processes, including changes in cardiac and breathing rates (Snyder et al., 1964). However, the data used in this work has undergone extensive clean-up using FIX in order to minimize the influence of structured noise associated with physiology. Therefore, the core findings presented in this work are unlikely to be purely driven by physiological fluctuations. Nevertheless, future research into the exact influence of physiological fluctuations on resting state amplitudes and on functional connectivity estimates would be of great interest.

When comparing the two scans acquired back-to-back on the same day, we found both increased amplitude of primary sensory/motor networks, as well as a large number of highly significant changes in functional connectivity between networks (in particular between pairs of two primary sensory/motor networks and for edges that included one primary sensory/motor and one cognitive network). Using an Additive Signal Change analysis, we confirmed that all of the observed changes in functional connectivity could be fully explained by the increased primary sensory/motor network amplitudes. This finding highlights the fact that changes in apparent functional connectivity can be observed in the absence of any changes in the coupling strength between two brain regions (e.g., changes in the relative timing of signal fluctuations, or in the strength of the direct neural coupling), instead being driven by signal amplitude and/or SNR changes (or by a shared connection to a third region, suggesting indirect coupling). These results are particularly pertinent to windowed connectivity measures, which are highly sensitive to the types of temporally dynamic changes in connectivity that we found in the HCP dataset. There is an important risk of misinterpreting such dynamic results as changes in functional coupling between regions, unless a windowed version of the ASC analysis approach is used, or dynamic changes in amplitude are explicitly investigated alongside changes in connectivity.

As briefly mentioned above, our findings point to a complex relationship between subject head motion and amplitude of both primary sensory/motor and default mode networks. Interestingly, correlations were particularly high between the slope of motion estimated within-subject, and the primary sensory/motor and DMN amplitude estimated across subjects, suggesting that motion may serve as a trait that varies across subjects in a potentially behaviourally relevant way. This is consistent with previous results that have shown that subject head motion is highly heritable (Couvy-Duchesne et al., 2014). A previous study has shown that between-subject differences in functional connectivity in the DMN between high-motion and low motion groups were not replicated when contrasting high-motion and low-motion scans within subjects, further supporting the notion that head motion may reflect a true between-subject trait instead of (or in addition to) being a simple source of artifactual noise (Zeng et al., 2014). Taken together, these results suggest that correlations with motion may (to some degree) represent real and interesting links, rather than being a purely artifactual influence; in light of this, further careful analysis that aims to disambiguate the former and the latter will be important.

In summary, the results presented in this work revealed previously unknown structured variation in resting state network amplitudes both across subjects and within subjects over time. The observed variability clearly distinguished between cognitive networks and early sensory and motor regions. This between-subject variability was replicated across both high and low dimensional network structures and in two large-scale independent datasets, suggesting that the observed differentiation between cognitive and early sensory/motor networks is a general feature of rfMRI data (although data with lower temporal resolution may result in more noisy amplitude estimates). Significant amplitude increases in sensory networks, possibly reflecting fluctuating arousal levels, were observed within subjects over relatively short timescales, and were shown to drive dynamic changes in functional connectivity. While these effects may be particularly prominent in acquisition protocols that include relatively long scan times (15 min in the HCP), significant changes were already observed between the first and second part of the first 7 min. Therefore, within-subject variability may be present in a large number of rfMRI datasets. Future rfMRI studies may benefit from obtaining arousal-related (self report) measures, and may wish to consider the influence of amplitude changes on measures of (dynamic) functional connectivity.
