# Supplementary material for: Investigations into within- and between-subject resting-state amplitude variations
Source: Neuroimage. 2017 Oct 1;159:57–69. doi: 10.1016/j.neuroimage.2017.07.014 (PMC5678294; doi:10.1016/j.neuroimage.2017.07.014)
Supplement: Supplementary file 1 [file mmc1.pdf]

Supplementary material and methods

Biobank sleep variables

Responses to sleep variables in UK Biobank data were obtained as part of a touch screen questionnaire performed on the same day that subjects completed the MRI scans. The following exact questions were used:

- Does your partner or a close relative or friend complain about your snoring? (Yes - No - Do not know - Prefer not to answer)
- Do you have a nap during the day? (Never/Rarely - Sometimes - Usually - Prefer not to answer)
- How likely are you to doze off or fall asleep during the daytime when you don't mean to? (eg: when working, reading or driving) (Never/rarely - Sometimes - Often - Do not know - Prefer not to answer)
- Do you have trouble falling asleep at night or do you wake up in the middle of the night? (Never/Rarely - Sometimes - Usually - Prefer not to answer)
- About how many hours sleep do you get in every 24 hours? (please include naps) (Enter number - Do not know - Prefer not to answer)
- On an average day, how easy do you find getting up in the morning? (Not at all easy - Not very easy - Fairly easy - Very easy - Do not know - Prefer not to answer)
- Do you consider yourself to be? (Definitely a 'morning' person - More a 'morning' than 'evening' person - More an 'evening' than a 'morning' person - Definitely an 'evening' person - Do not know - Prefer not to answer)

Processing of the sleep measures was performed in an analogous way to the HCP behavioural variables described above.

Additive Signal Change (ASC) analysis

The ASC analysis determines whether changes in correlation might be explained by simple increases in signal amplitude in one run. Assuming increases in amplitude occur in run 2, we model the changes in signal as:

$X_1 = Sx$

$Y_1 = Sy$

$X_2 = Sx + Nx$
$Y_2 = Sy + Ny$

$corr(S_1,N_1) > 0$
$corr(S_2,N_2) > 0$

Given observables $std(X_1)$, $std(X_2)$, $std(Y_1)$, $std(Y_2)$, $corr(X_1,Y_1)$, we can calculate bounds for $corr(X_2,Y_2)$ given the above model. Observation uncertainty can be integrated into these calculations. Full details of the ASC analysis can be found in (Duff, Makin, Smith, & Woolrich, 2017).

Supplementary results

Excluding the role of phase-encode direction on amplitude changes

Data for the HCP were acquired using two different phase encode directions, such that on each day on of the scans was acquired left to right, and the other scan was acquired right to left. To exclude the possibility that the within-subject results across runs presented in figure 4A in the main manuscript were explained by phase encode direction we ran a test on a subset of subjects. We selected 30 subjects for whom the first scan on day 2 used right-left (RL) phase encoding (followed by left-right as the second scan), and 30 subjects for whom the first scan on day 2 used left-right (LR) followed by the right-left scan (the order of the phase encode scans were only randomised at a later stage in the HCP data collection, so only 30 subjects were available in the right-left followed by left-right order). Within each subject, primary sensory/motor amplitudes were averaged across the 14 ICs in the primary sensory/motor cluster for run 1 and run 2. A repeated measures ANOVA with one within-subject factor for scan (first or second) and one between-subject factor for order (LR or RL first) was performed. The results show a significant main effect of the within-subject factor of scan ($F(1,58)=10.2$, $p=0.002$), in line with the findings presented. The main effect of the between-subject factor for order is not significant ($F(1,58)=0.8$, $p=0.37$), and the interaction effect is also not significant ($F(1,58)=0.2$, $p=0.66$).

Therefore, we do not find any evidence suggesting that the phase encode direction plays a role in the observed increases in amplitude.

References

Duff, E. P., Makin, T., Smith, S. M., & Woolrich, M. W. (2017, January 25). *Disambiguating brain functional connectivity*. *bioRxiv*. https://doi.org/10.1101/103002

Hotelling, H. (1936). Relations between two sets of variates. *Biometrika*, *28*(3-4), 321–377. https://doi.org/10.1093/biomet/28.3-4.321

Smith, S. M., Nichols, T. E., Vidaurre, D., Winkler, A. M., Behrens, T. E. J., Glasser, M. F., … Miller, K. L. (2015). A positive-negative mode of population covariation links brain connectivity, demographics and behavior. *Nature Neuroscience*. https://doi.org/10.1038/nn.4125

Winkler, A. M., Webster, M. A., Vidaurre, D., Nichols, T. E., & Smith, S. M. (2015). Multi-level block permutation. *NeuroImage*, *123*, 253–268. https://doi.org/10.1016/j.neuroimage.2015.05.092
