# Supplementary material for: Investigations into within- and between-subject resting-state amplitude variations
Source: Neuroimage. 2017 Oct 1;159:57–69. doi: 10.1016/j.neuroimage.2017.07.014 (PMC5678294; doi:10.1016/j.neuroimage.2017.07.014)
Supplement: Supplementary file 2 [file mmc2.pdf]

Figure S1: Cross-subject clustering result for high dimensional ICA (d=200)

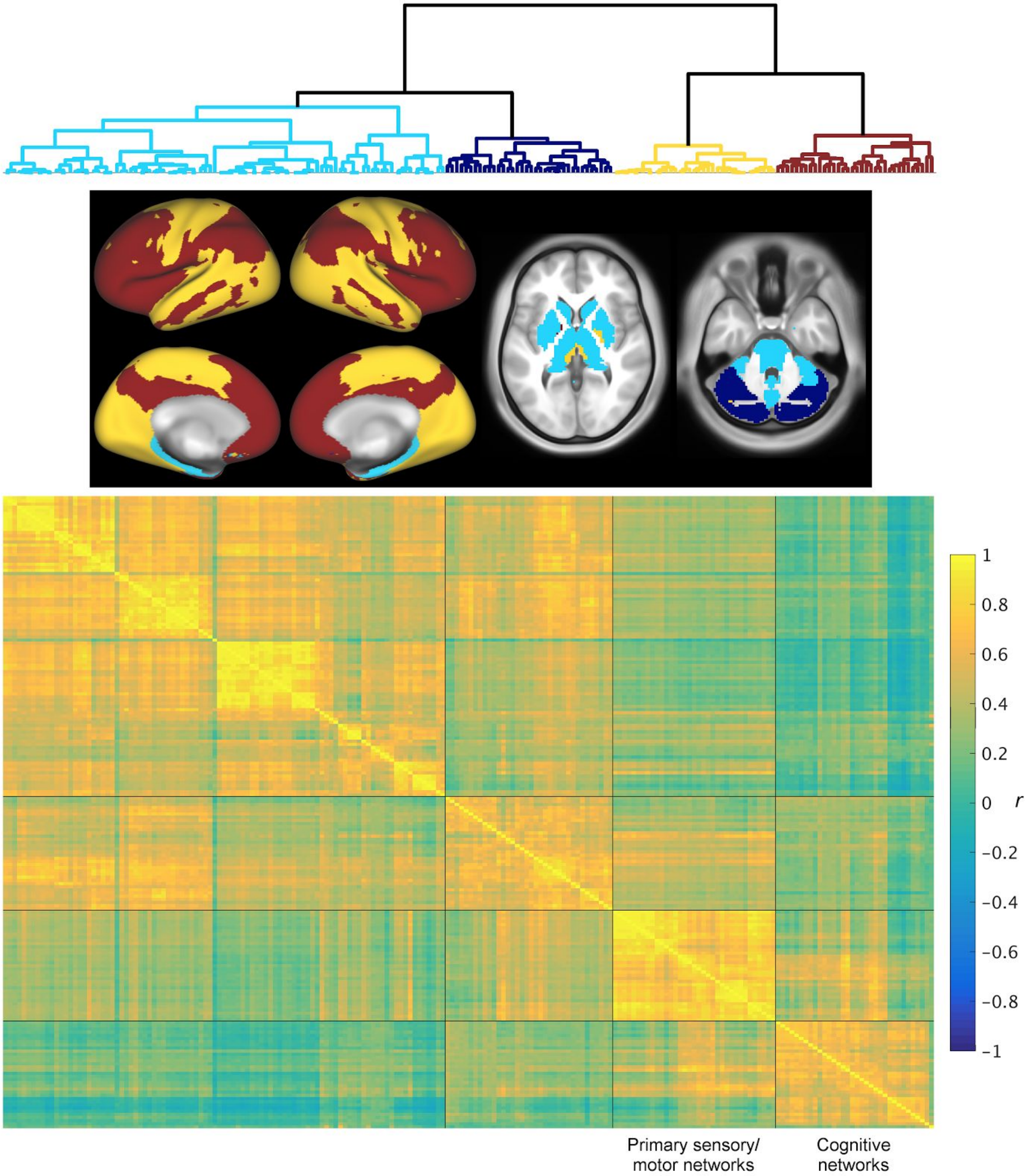

Figure S1: Between subject clustering of network amplitude correlation matrix was repeated for 200-dimensionality group-ICA networks. The clustering results at this higher dimensionality replicated the 25-dimensionality results presented in figure 1, as cortical components were split into primary sensory/motor versus cognitive networks (in addition to a further split between cortical and subcortical components).

Figure S2: Correlations between two CCA modes and behavioural measures

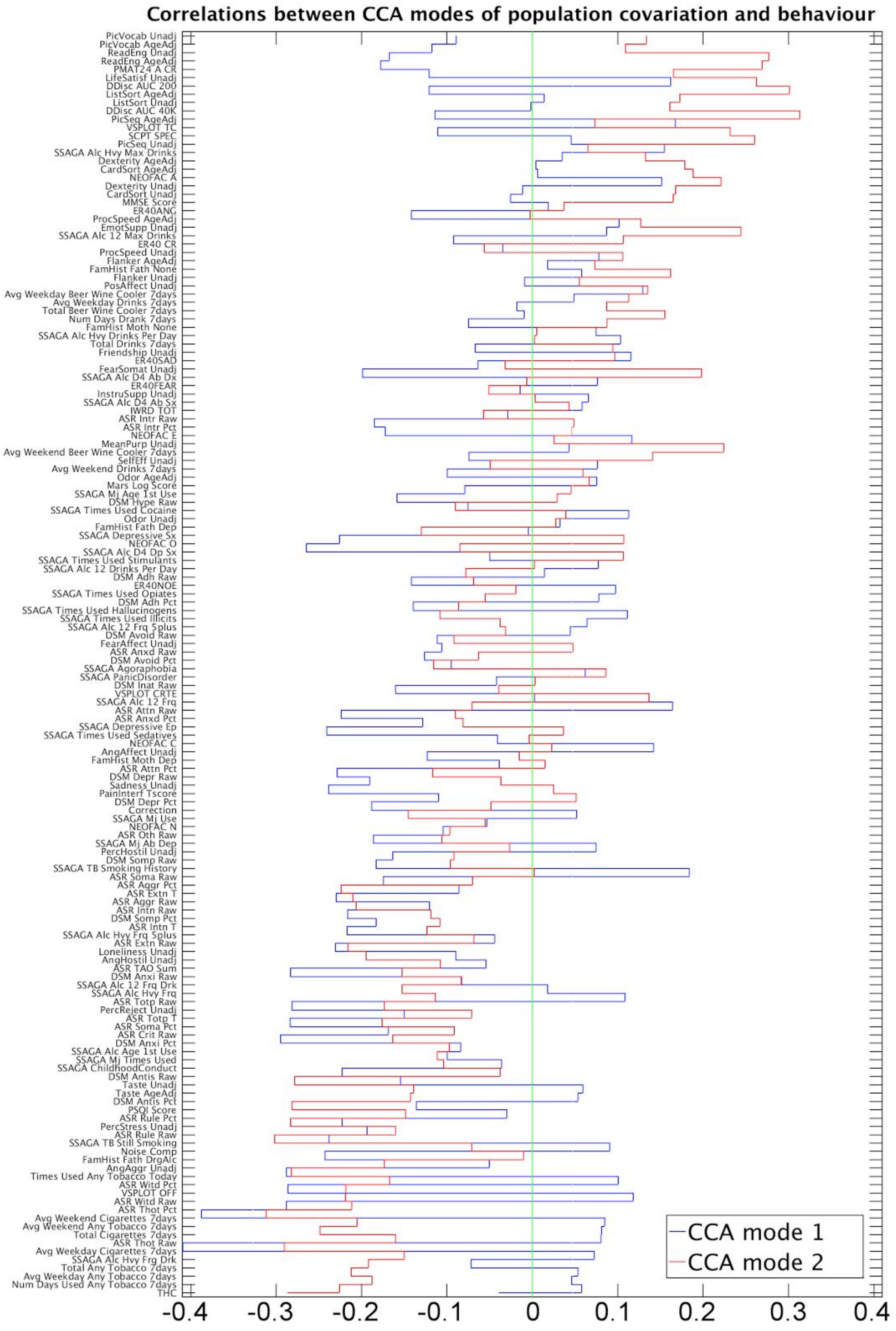
Correlations between CCA modes of population covariation and behaviour
PicVocab Unadj
PicVocab AgeAdj
ReadEng Unadj
ReadEng AgeAdj
PMAT24 A CR
LifeSatisf Unadj
DDisc AUC 200
ListSort AgeAdj
ListSort Unadj
DDisc AUC 40K
PicSeq AgeAdj
VSPLOT TC
SCPT SPEC
PicSeq Unadj
SSAGA Alc Hvy Max Drinks
Dexterity AgeAdj
CardSort AgeAdj
NEOFAC A
Dexterity Unadj
CardSort Unadj
MMSE Score
ER40ANG
ProcSpeed AgeAdj
EmotSupp Unadj
SSAGA Alc 12 Max Drinks
ER40 CR
ProcSpeed Unadj
Flanker AgeAdj
FamHist Fath None
Flanker Unadj
PosAffect Unadj
Avg Weekday Beer Wine Cooler 7days
Avg Weekday Drinks 7days
Total Beer Wine Cooler 7days
Num Days Drank 7days
FamHist Moth None
SSAGA Alc Hvy Drinks Per Day
Total Drinks 7days
Friendship Unadj
ER40SAD
FearSomat Unadj
SSAGA Alc D4 Ab Dx
ER40FEAR
InstruSupp Unadj
SSAGA Alc D4 Ab Sx
IWRD TOT
ASR Intr Raw
ASR Intr Pct
NEOFAC E
MeanPurp Unadj
Avg Weekend Beer Wine Cooler 7days
SelfEff Unadj
Avg Weekend Drinks 7days
Odor AgeAdj
Mars Log Score
SSAGA Mj Age 1st Use
DSM Hype Raw
SSAGA Times Used Cocaine
Odor Unadj
FamHist Fath Dep
SSAGA Depressive Sx
NEOFAC O
SSAGA Alc D4 Dp Sx
SSAGA Times Used Stimulants
SSAGA Alc 12 Drinks Per Day
DSM Adh Raw
ER40NOE
SSAGA Times Used Opiates
DSM Adh Pct
SSAGA Times Used Hallucinogens
SSAGA Times Used Illicits
SSAGA Alc 12 Frq 5plus
DSM Avoid Raw
FearAffect Unadj
ASR Anxd Raw
DSM Avoid Pct
SSAGA Agoraphobia
SSAGA PanicDisorder
DSM Inat Raw
VSPLOT CRTE
SSAGA Alc 12 Frq
ASR Attn Raw
ASR Anxd Pct
SSAGA Depressive Ep
SSAGA Times Used Sedatives
NEOFAC C
AngAffect Unadj
FamHist Moth Dep
ASR Attn Pct
DSM Depr Raw
Sadness Unadj
PainInterf Tscore
DSM Depr Pct
Correction
SSAGA Mj Use
NEOFAC N
ASR Oth Raw
SSAGA Mj Ab Dep
PercHostil Unadj
DSM Somp Raw
SSAGA TB Smoking History
ASR Soma Raw
ASR Aggr Pct
ASR Extn T
ASR Aggr Raw
ASR Intn Raw
DSM Somp Pct
ASR Intn T
SSAGA Alc Hvy Frq 5plus
ASR Extn Raw
Loneliness Unadj
AngHostil Unadj
ASR TAO Sum
DSM Anxi Raw
SSAGA Alc 12 Frq Drk
SSAGA Alc Hvy Frq
ASR Totp Raw
PercReject Unadj
ASR Totp T
ASR Soma Pct
ASR Crit Raw
DSM Anxi Pct
SSAGA Alc Age 1st Use
SSAGA Mj Times Used
SSAGA ChildhoodConduct
DSM Antis Raw
Taste Unadj
Taste AgeAdj
DSM Antis Pct
PSQI Score
ASR Rule Pct
PercStress Unadj
ASR Rule Raw
SSAGA TB Still Smoking
Noise Comp
FamHist Fath DrgAlc
AngAggr Unadj
Times Used Any Tobacco Today
ASR Witd Pct
VSPLOT OFF
ASR Witd Raw
ASR Thot Pct
Avg Weekend Cigarettes 7days
Avg Weekend Any Tobacco 7days
Total Cigarettes 7days
ASR Thot Raw
Avg Weekday Cigarettes 7days
SSAGA Alc Hvy Frg Drk
Total Any Tobacco 7days
Avg Weekday Any Tobacco 7days
Num Days Used Any Tobacco 7days
THC
-0.4
-0.3
-0.2
-0.1
0
0.1
0.2
0.3
0.4
CCA mode 1
CCA mode 2

Figure S2: The first two significant CCA modes of population covariation were correlated back into each of the behavioural measures (on the y-axis, ordered based on their correlation with the original positive-negative mode of covariation in Smith et al., 2015).

Figure S3: Within-run changes using fALFF as a measure of amplitude

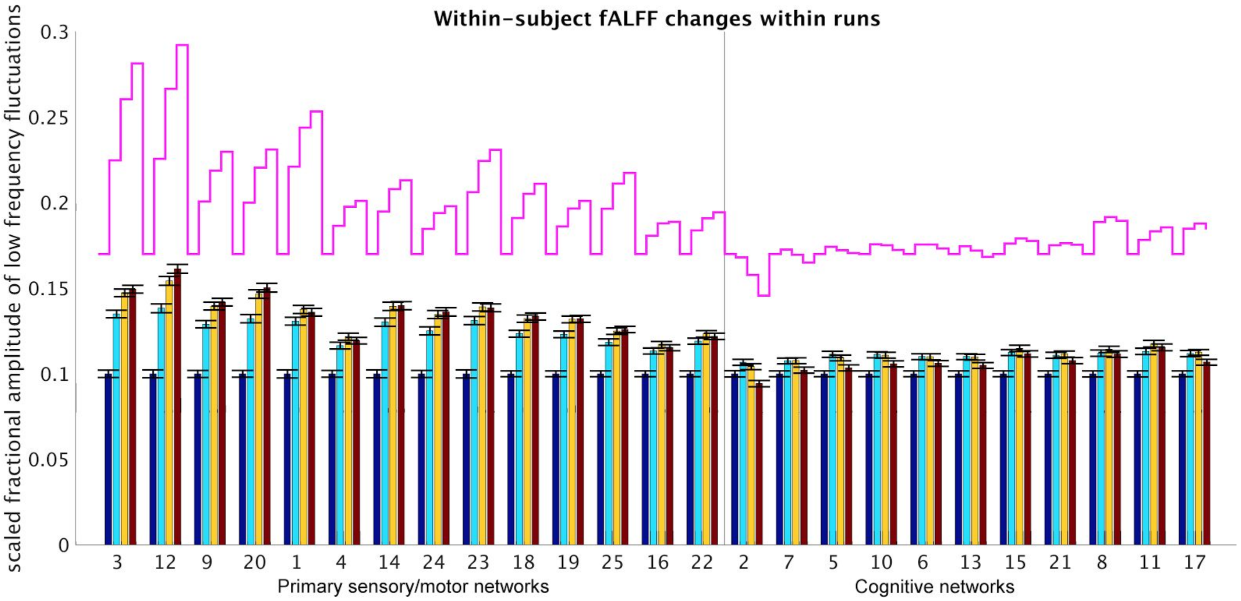

Figure S3: This figure shows the same results as figure 4B but using fALFF as a measure of amplitude instead of the timeseries standard deviation. To allow easy comparison of within-run changes, fALFF results were scaled within each network by removing the amplitude in the first ¼ from all 4 bars and adding 0.1. Lines in magenta show the scaled means from figure 4B using amplitudes to facilitate comparison. The results show that the same characteristic within-run increases in primary sensory/motor networks can be observed using fALFF.

Figure S4: Statistical comparison of between-run and within-run changes in amplitude

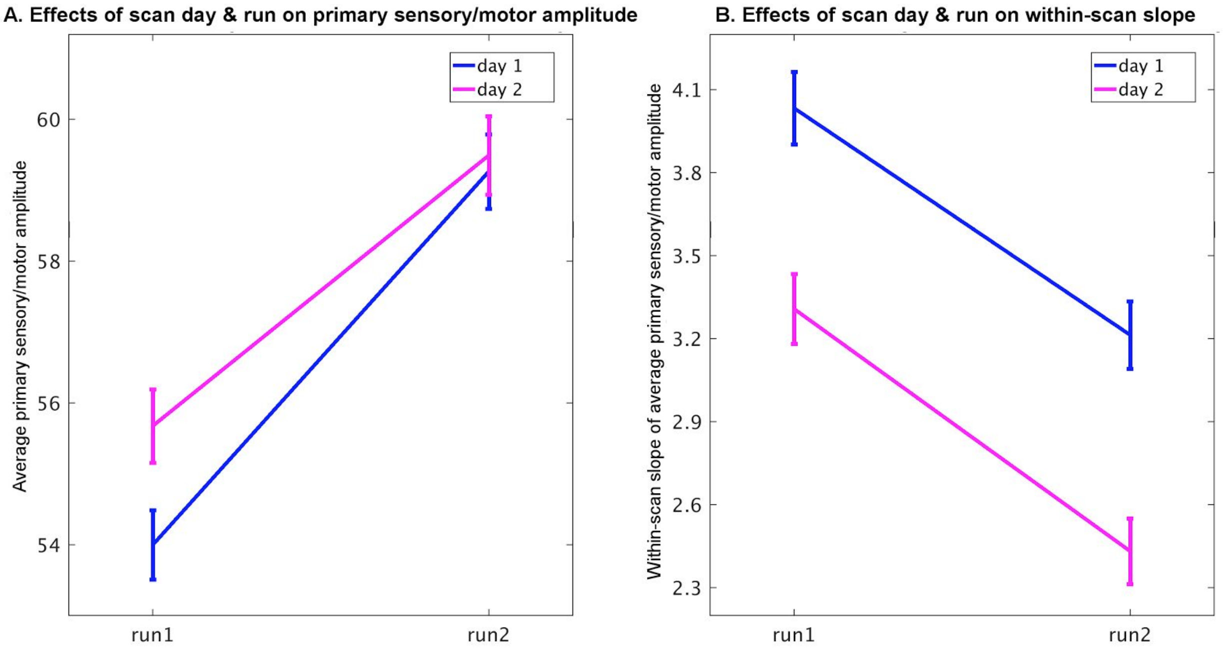

Figure S4: Statistical comparison of the effects of day and run on both between-run changes (A) and within-run changes (B) in amplitude. The findings show that primary sensory/motor amplitudes were significantly lower at the start of day one, compared with the start of day 2. Within each run, there was an increase in primary sensory/motor amplitudes from the start to the end of the run, but the slope of this increase was steeper during run 1 compared with run 2 on the same day.

Figure S5: Frequency components of within run changes

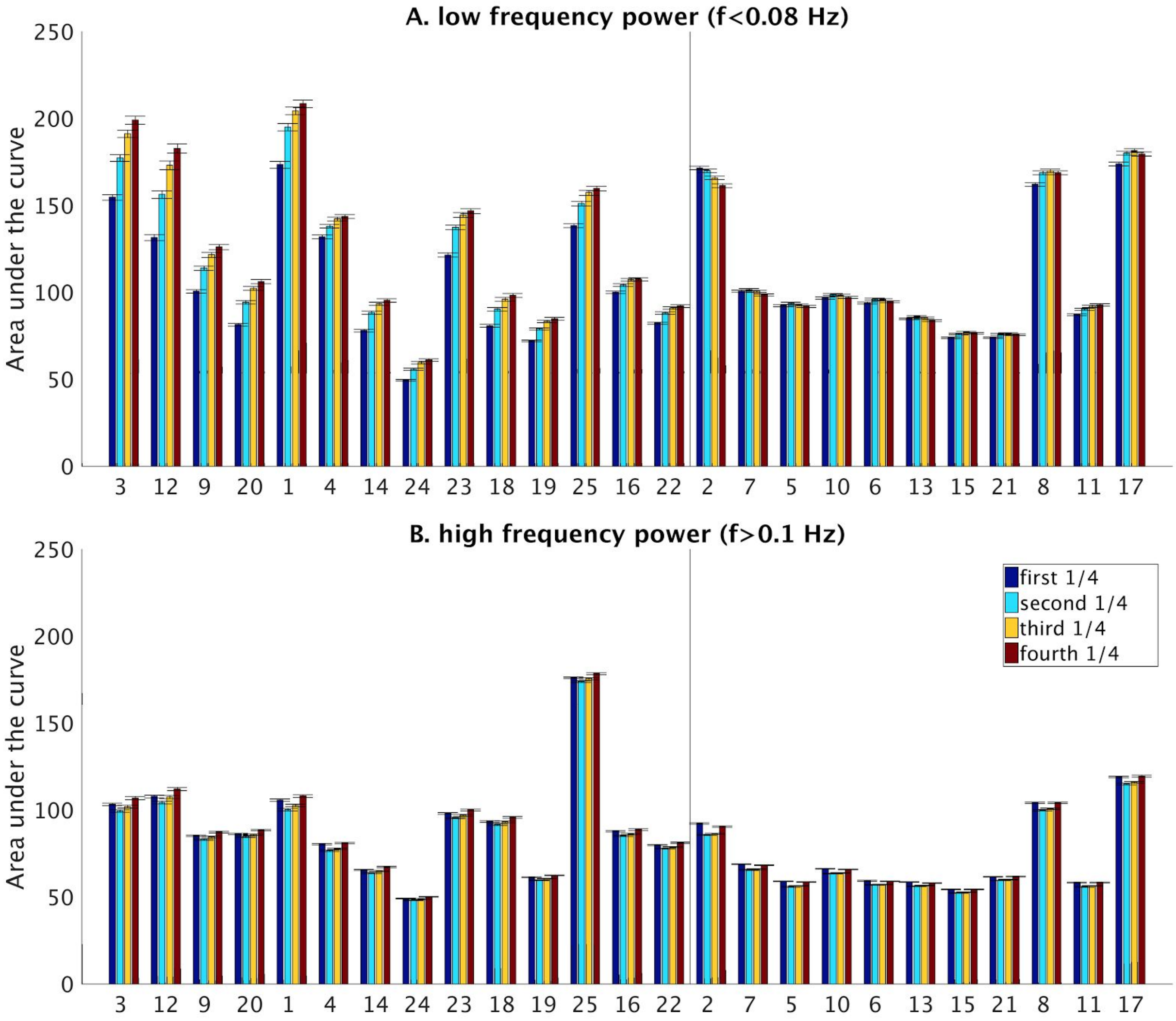

Figure S5: Within-run changes in low and high frequency power. Fourier transforms were calculated for each 4-minute block for each subject, and areas under the curve were estimated for the low frequency range (<0.08 Hz) and for the high frequency range (>0.1 Hz). The barplots show the average area under the curve across subjects and error bars indicate the standard error of the mean. These results show that the increase in amplitude observed across the runs is associated with increased low frequency amplitude, suggesting that it may be neuronal in origin.

Figure S6: Within run changes after volume censoring

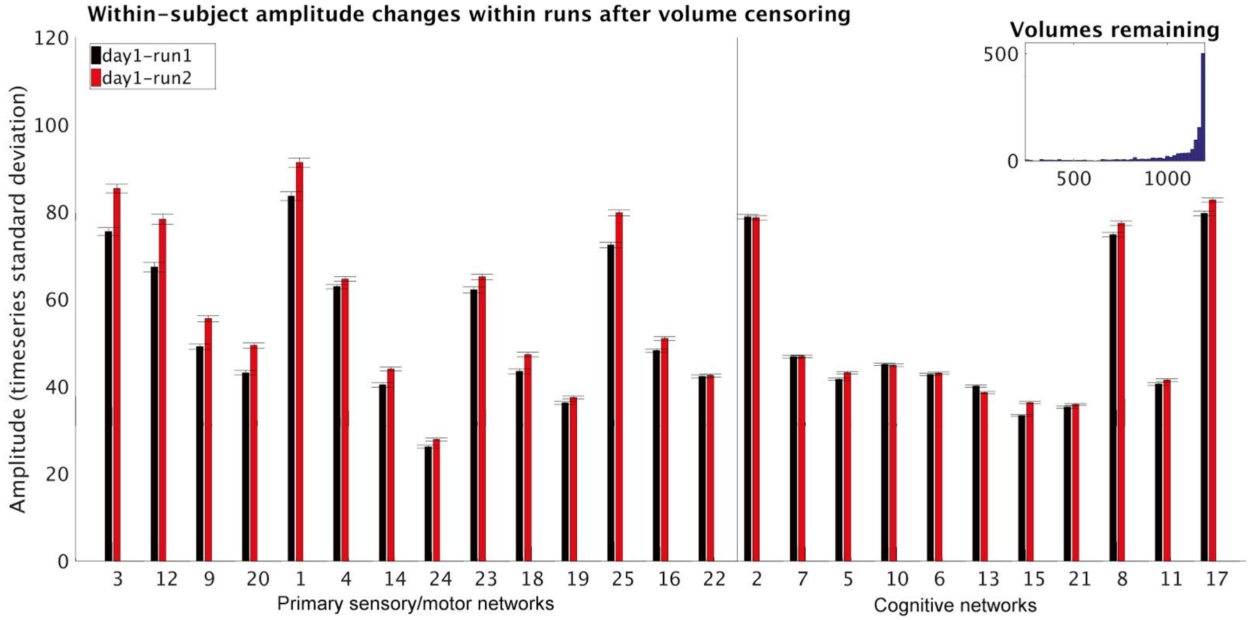

Figure S6: After volume censoring is performed on data from day 1, the same increase in BOLD amplitude of primary sensory/motor networks in run two compared to run one is seen. The figure insert in the top right corner shows the number of volumes retained after volume censoring (scans with fewer than 240 volumes retained were excluded from this analysis). This confirms that these findings are not driven by motion-induced artefacts.

Figure S7: Cross-subject clustering results for UK Biobank (ICA d = 25)

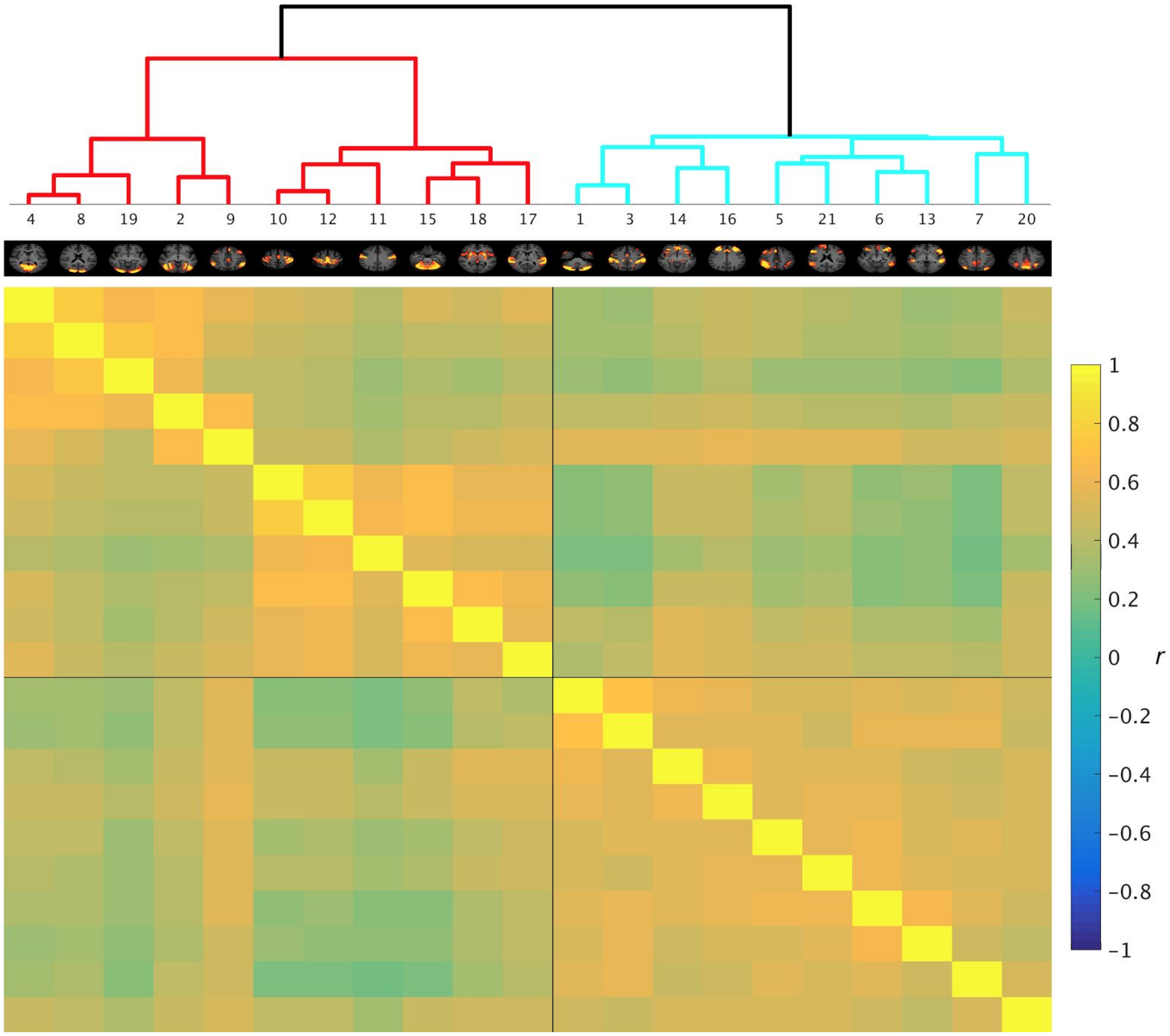

Figure S7: Cross subject clustering of network amplitude correlation matrix in the UK Biobank data. The cluster tree at the top clearly separates the networks into two distinct clusters, and these clusters are very similar to the clusters shown in figure 1 with primary sensory/motor networks in the red cluster on the left and cognitive networks in the blue cluster on the right.

Figure S8: Changes in functional connectivity between run 1 on day 1 and run 1 on day 2

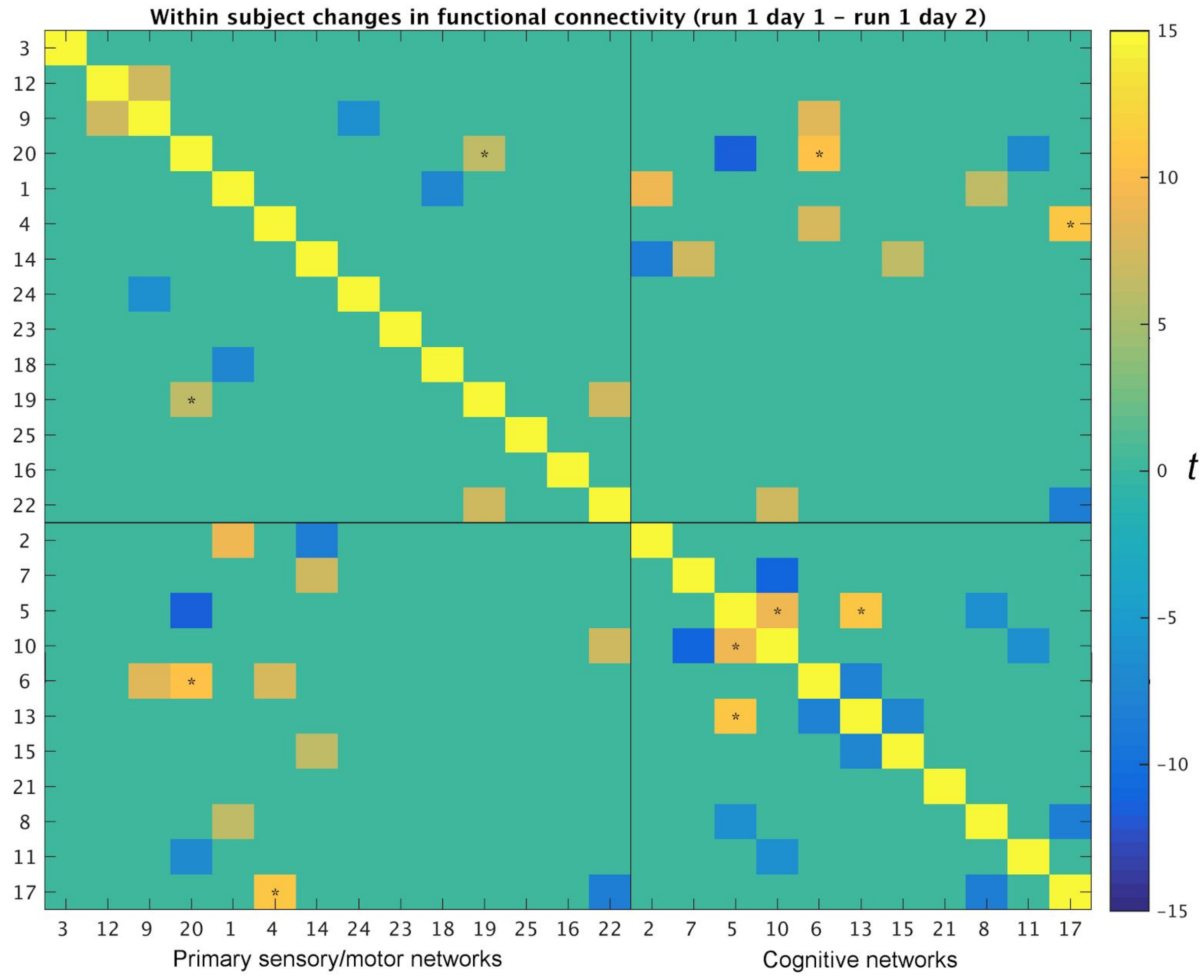

*Figure S8: Changes in functional connectivity occurring as a result of amplitude changes. The colour scale reflects t statistics for the paired t-test between run 1 on day 1 and run 1 on day 2 (only edges that pass Bonferroni correction and have a $|t|>6$ are shown). These changes in functional connectivity could only partially be explained by changes in network amplitude. Cases where additive signal common to both networks explained the change in functional connectivity are marked *.*
